# Author Correction: Accelerometer-derived physical activity and mortality in individuals with type 2 diabetes

**DOI:** 10.1038/s41467-024-54119-y

**Published:** 2024-11-18

**Authors:** Zhi Cao, Jiahao Min, Han Chen, Yabing Hou, Hongxi Yang, Keyi Si, Chenjie Xu

**Affiliations:** 1 School of Public Health, Hangzhou Normal University, Hangzhou, China; 2 School of Public Health, Zhejiang University School of Medicine, Hangzhou, China; 3 Yanjing Medical College, Capital Medical University, Beijing, China; 4 School of Basic Medical Sciences, Tianjin Medical University, Tianjin, China; 5 School of Public Health, Shanghai Jiao Tong University School of Medicine, Shanghai, China

**Keywords:** Risk factors, Type 2 diabetes, Public health

**Correction to:** *Nature Communications* 10.1038/s41467-024-49542-0, published online 17 June 2024

The original version of this article included errors in the Results, Discussion, and Supplementary Information. During analysis of identifying individuals with type 2 diabetes, a mistake in the codes has led to an overestimation of the sample size. The error occurred due to the default setting of the STATA 16.0 software, which assigns missing values as positive infinity when defining diabetes using HbA1c ≥ 48 mmol/mol, resulting in the inclusion of individuals with missing blood glucose data and an inflated sample size.

The relevant codes have now been rechecked and the data has now been reanalyzed. The sample size of this study is now revised as 4003 patients with type 2 diabetes. The primary findings and conclusions are not affected. The following changes have been made in the text and figures:

The sentence that read “Here, we perform a prospective cohort study of 19,624 individuals with type 2 diabetes (T2D) from the UK Biobank with a median follow-up of 6.9 years”, now reads “Here, we perform a prospective cohort study of 4003 individuals with type 2 diabetes (T2D) from the UK Biobank with a median follow-up of 6.9 years”.

The sentence that read “12.7%, 15.8%, and 22.3% of deaths are attributable to the lowest level of light-intensity, moderate-intensity PA, and vigorous-intensity PA, respectively.”, now reads “18.8%, 28.0%, and 31.1% of deaths are attributable to the lowest level of light-intensity PA, moderate-intensity PA, and vigorous-intensity PA, respectively.”.

The sentence that read “As shown in Supplementary Fig. 1, of the 502,401 UK biobank participants, 118,381 had T2D at baseline, of whom 22,309 had accelerometry data.”, now reads “As shown in Supplementary Fig. 1, of the 502,401 UK biobank participants, 32,709 had T2D at baseline, of whom 4604 had accelerometry data.”.

The sentence that read “After excluding those who had insufficient wear time (n = 1538), daylight saving time shifts during wear period (n = 909), and missing information on covariates (n = 238), 19,624 participants with T2D were left in the main analysis, with a mean age of 62.3 years (standard deviation, 7.8 years) and 44.4% were males.”, now reads “After excluding those who had insufficient wear time (n = 312), daylight saving time shifts during wear period (n = 174), and missing information on covariates (n = 115), 4003 participants with T2D were left in the main analysis, with a mean age of 64.9 years (standard deviation, 6.9 years) and 63.0% were males.”.

The sentence that read “During a median follow-up of 6.9 years (interquartile range 6.4–7.4 years), 810 death cases were documented.”, now reads “During a median follow-up of 6.9 years (interquartile range 6.3–7.4 years), 339 death cases were documented.”.

The sentence that read “Regardless of PA intensity, the dose-response associations of PA duration with all-cause mortality were L-shaped (p value for non-linearity <0.001, Fig. 2).”, now reads “Regardless of PA intensity, the dose-response associations of PA duration with all-cause mortality were L-shaped (Fig. 2).”.

The sentence that read “At the inflection points, associations were observed with all-cause mortality reductions of ~60% at 1800 min/week for LPA, 70% at 400 min/week for moderate-intensity PA (MPA), 45% at 20 min/week for VPA, and 70% at 400 min/week for MVPA, compared with the least active one percentile.”, now reads “At the inflection points, associations were observed with all-cause mortality reductions of ~60% at 1800 min/week for LPA, 70% at 300 min/week for moderate-intensity PA (MPA), 45% at 30 min/week for VPA, and 70% at 300 min/week for MVPA, compared with the least active one percentile.”.

The sentence that read “Additional associations of all-cause mortality risk reductions followed by a longer duration of PA were limited for LPA (additional 5% by as long as 3000 min/week) but significant for MPA (20% by 1200 min/week), VPA (40% by 200 min/week), and MVPA (20% by 1200 min/week).”, now reads “Additional associations of all-cause mortality risk reductions followed by a longer duration of PA were limited for VPA (additional 1% by as long as 100 min/week) but significant for LPA (15% by 3000 min/week), MPA (20% by 1000 min/week), and MVPA (20% by 1000 min/week).”.

The sentence that read “For example, compared with <150 min/week of MPA, the multivariable-adjusted hazard ratios (HRs) and 95% confidence intervals (CIs) for 150–299, 300–449, and ≥450 min/week were 0.57 (0.47–0.70), 0.42 (0.34–0.53), and 0.32 (0.25–0.40), respectively.”, now reads “For example, compared with <150 min/week of MPA, the multivariable-adjusted hazard ratios (HRs) and 95% confidence intervals (CIs) for 150–299, 300–449, and ≥450 min/week were 0.61 (0.47–0.79), 0.41 (0.29–0.56), and 0.24 (0.15–0.36), respectively.”.

The sentence that read “For example, the population-attributable fractions (PAFs) for mortality sequentially decreased with higher durations of MVPA, with PAFs of 28.9%, 6.3%, and 3.4% for those engaging <275 min/week, 275–450 min/week, and 450–624 min/week, respectively.”, now reads “For example, the population-attributable fractions (PAFs) for mortality sequentially decreased with higher durations of MVPA, with PAFs of 47.1%, 8.8%, and 1.0% for those engaging <275 min/week, 275–449 min/week, and 450–624 min/week, respectively.”.

The sentence that read “Similar patterns to MVPA were observed for other PA intensities, with 12.7%, 15.8%, and 22.3% of PAFs for the lowest activity groups of LPA, MPA, and VPA, respectively.”, now reads “Similar patterns to MVPA were observed for other PA intensities, with 18.8%, 28.0%, and 31.1% of PAFs for the lowest activity groups of LPA, MPA, and VPA, respectively.”.

The sentence that read “A total of 441 cancer deaths (54.4% of all deaths) and 172 CVD deaths (21.2% of all deaths) were recorded.”, now reads “A total of 158 cancer deaths (46.6% of all deaths) and 83 CVD deaths (24.5% of all deaths) were recorded.”.

The sentence that read “Compared with <150 min/week of MPA, ≥450 min/week of MPA was associated with a 56% (95% CI: 39%–69%) and 55% (95% CI: 23%–72%) lower risk of cancer and CVD mortality, respectively.”, now reads “Compared with <150 min/week of MPA, ≥450 min/week of MPA was associated with a 63% (95% CI: 33%–80%) and 68% (95% CI: 21%–87%) lower risk of cancer and CVD mortality, respectively.”.

The sentence that read “Meanwhile, the HRs with VPA ≥ 30 min/week were 0.58 (95% CI: 0.42–0.81) for cancer mortality, and 0.28 (95% CI: 0.14–0.55) for CVD-cause mortality, compared with none of VPA.”, now reads “Meanwhile, the HRs with VPA ≥ 30 min/week were 0.61 (95% CI: 0.32–1.17) for cancer mortality, and 0.35 (95% CI: 0.10–1.15) for CVD-cause mortality, compared with none of VPA.”.

The sentence that read “Different combinations of PA could associate with similar risk reductions in all-cause mortality, such as 150–299 min/week of MPA and 2100–2449 min/week of LPA (HR = 0.50; 95% CI: 0.35–0.72), 1–14 min/week of VPA and ≥2450 min/week of LPA (HR = 0.52; 95% CI: 0.36–0.76), 1–14 min/week of VPA and 150–299 min/week of MPA (HR = 0.50; 95% CI: 0.38–0.65).”, now reads “Different combinations of PA could associate with similar risk reductions in all-cause mortality, such as 300–449 min/week of MPA and 2100–2449 min/week of LPA (HR = 0.35; 95% CI: 0.19–0.63), 1–14 min/week of VPA and 2100–2449 min/week of LPA (HR = 0.33; 95% CI: 0.17–0.63), 1–14 min/week of VPA and 300–449 min/week of MPA (HR = 0.35; 95% CI: 0.22–0.54).”.

The sentence that read “No statistically significant interactions were found between all PA intensities and age, BMI, waist circumference, smoking status, alcohol intake, diet scores, sleep scores, and history of hypertension, except sex. (p value for interaction > 0.05, Supplementary Tables 11–14). The association between LPA and all-cause mortality was stronger among female than male (p value for interaction = 0.031).”, now reads “No statistically significant interactions were found between all PA intensities and age, sex, BMI, waist circumference, smoking status, alcohol intake, diet scores, sleep scores, and history of hypertension (p value for interaction > 0.05, Supplementary Tables 11-14).”.

The sentence that read “In this prospective cohort study of 19,624 adults with T2D, we found that irrespective of PA intensity, longer duration of accelerometer-measured PA was significantly associated with a lower risk of all-cause, cancer, and CVD mortality, without minimal or maximal threshold. Up to 38.6% deaths among T2D patients could potentially be associated with performing <625 min/week of MVPA.”, now reads “In this prospective cohort study of 4003 adults with T2D, we found that irrespective of PA intensity, longer duration of accelerometer-measured PA was significantly associated with a lower risk of all-cause, cancer, and CVD mortality, without minimal or maximal threshold. Up to 56.9% deaths among T2D patients could potentially be associated with performing <625 min/week of MVPA.”.

The sentence that read “In our study, a 70% of risk reduction in all-cause mortality was seen at the inflection point of MVPA (400 min/week), larger than the 55% at 500 min/week by self-report in a US study of 41,726 participants diabetic adults21.”, now reads “In our study, a 70% of risk reduction in all-cause mortality was seen at the inflection point of MVPA (300 min/week), larger than the 55% at 500 min/week by self-report in a US study of 41,726 diabetic adults21.”.

The sentence that read “Moreover, we found that every 600 MET-min/week increase of accelerometer-measured PA was associated with 8% lower risk of all-cause mortality in patients with T2D, much larger than 4% as reported by a meta-analysis of six prospective cohort studies (N = 32,221)22. Similarly, 51% or 48% risk reduction of all-cause mortality was associated with ≥150 min/week of MPA or ≥75 min/week of VPA in our study, much larger than 14% from another study using self-reported PA in the UK Biobank9.”, now reads “Moreover, we found that every 600 MET-min/week increase of accelerometer-measured PA was associated with 11% lower risk of all-cause mortality in patients with T2D, much larger than 4% as reported by a meta-analysis of six prospective cohort studies (N = 32,221)22. Similarly, 50% risk reduction of all-cause mortality was associated with ≥150 min/week of MPA in our study, much larger than 14% from another study using self-reported PA in the UK Biobank9.”.

The sentence that read “The L-shaped dose-response associations of accelerometer-measured LPA and MVPA with all-cause mortality in T2D patients in our study mirrored those in the general population in a harmonized meta-analysis, with similar inflection points for LPA (around 2000 min/week) but different ones for MVPA (T2D: 400 min/week; general: 140 min/week)14.”, now reads “The L-shaped dose-response associations of accelerometer-measured LPA and MVPA with all-cause mortality in T2D patients in our study mirrored those in the general population in a harmonized meta-analysis, with similar inflection points for LPA (around 2000 min/week) but different ones for MVPA (T2D: 300 min/week; general: 140 min/week)14.”.

The sentence that read “The magnitude of risk reduction was smaller in T2D patients than in the general from 1400 min/week to 2100 min/week of LPA (33% vs. 50%) and from around 0 to 140 min/week of MVPA (50% vs. 60%), but beyond this level, the risk continued to decrease in T2D patients while levelled off or even slightly increased in the general.”, now reads “The magnitude of risk reduction was smaller in T2D patients than in the general from 1400 min/week to 2100 min/week of LPA (26% vs. 50%) and from around 0 to 140 min/week of MVPA (55% vs. 60%), but beyond this level, the risk continued to decrease in T2D patients while levelled off or even slightly increased in the general.”.

The sentence that read “In our study, the risk for all-cause mortality decreased sharply as the duration of MPA increased to 400 min/week and then became steady till around 600 min/week before another drop began. A dramatic risk reduction was also seen in VPA till 20 min/week, with a less drastic magnitude thereafter.”, now reads “In our study, the risk for all-cause mortality decreased sharply as the duration of MPA increased to 300 min/week with a less drastic magnitude thereafter. A dramatic risk reduction was also seen in VPA till 30 min/week and then became steady.”.

The sentence that read “This finding suggests that the effort required to increase the volume of MPA may not be proportional to the benefit after reaching 400 min/week, while the recommended threshold of VPA can be lowered to 20 min/week, which can bring considerable benefit and is easier to implement.”, now reads “This finding suggests that the effort required to increase the volume of MPA may not be proportional to the benefit after reaching 300 min/week, while the recommended threshold of VPA can be lowered to 30 min/week, which can bring considerable benefit and is easier to implement.”.

The sentence that read “In this study, participants with T2D at baseline were identified through an integration of multiple data sources: self-reported T2D, measured random glucose level ≥11.1 mmol/L or glycated hemoglobin (HbA1c) level ≥48 mmol/L according to the American Diabetes Association criteria, and code E11 in the electronic health records (England and Wales: Health Episode Statistics; Scotland: Scottish Morbidity Records) according to the 10th Revision of the International Classification of Diseases (ICD-10).”, now reads “In this study, participants with T2D at baseline were identified through an integration of multiple data sources: self-reported T2D, insulin use or glycated hemoglobin (HbA1c) level ≥48 mmol/mol according to the American Diabetes Association criteria, and code E11 in the electronic health records (England and Wales: Health Episode Statistics; Scotland: Scottish Morbidity Records) according to the 10th Revision of the International Classification of Diseases (ICD-10).”.

The sentence that read “the inflection points of the dose-response relationship in this study (about 1750, 400, 15, and 375 min/week for LPA, MPA, VPA, and MVPA)”, now reads “the inflection points of the dose-response relationship in this study (about 1750, 300, 30, and 275 min/week for LPA, MPA, VPA, and MVPA)”.

The legend of Fig. 1 that read “The study includes 19,624 participants with type 2 diabetes from the UK Biobank to explore the association between accelerometer-derived physical activity and all-cause and cause-specific mortality.”, now reads “The study includes 4003 participants with type 2 diabetes from the UK Biobank to explore the association between accelerometer-derived physical activity and all-cause and cause-specific mortality.”.

The legend of Fig. 3 that read “We utilized diabetic participants (n = 19,624) from the UK Biobank with valid accelerometer data in the analyses.”, now reads “We utilized diabetic participants (n = 4003) from the UK Biobank with valid accelerometer data in the analyses.”.

The legend of Fig. 4 that read “We utilized diabetic participants (n = 19,624) from the UK Biobank with valid accelerometer data in the analyses.”, now reads “We utilized diabetic participants (n = 4003) from the UK Biobank with valid accelerometer data in the analyses.”.

The original version of the Supplementary Information associated with this Article contained errors in the Methods, Supplementary Tables 1–27, and Supplementary Figs. 1–3. The HTML has been updated to include a corrected version of the Supplementary Information. The original incorrect versions of Methods, these Figures and Tables can be found as Supplementary Information associated with this Correction.

In Supplementary Methods, the numbers to depict missing proportion of covariates were changed. The sentence that read ‘Alcohol intake (missing proportion: 0.09%), smoking status (0.31%), diet scores (5.21%), sleep scores (0.06%), self-rated health status (0.31%), long-standing illness, disability or infirmity (1.88%), illness, injury, bereavement, stress in last 2 years (2.86%).‘ now reads ‘Ethnicity (0.36%), education (1.29%), BMI (0.53%), waist (0.27%), alcohol intake (missing proportion: 0.01%), smoking status (0.39%), diet scores (4.18%), diabetes duration (0.01%), self-rated health status (0.46%), long-standing illness, disability or infirmity (1.80%), illness, injury, bereavement, stress in last 2 years (0.97%).

In the Supplementary Tables 1–4, the numbers to show baseline characteristics of participants were changed. All numbers for HRs (95% CIs) and PAFs (95% CIs) in the Supplementary Tables 5–26 were changed. The P-value for the proportional hazard test, as presented in the Supplementary Table 27, has been correspondingly corrected.

The number of individuals for every step of exclusion in Supplementary Fig. 1 “118,381, 98,699, 96,072, 1538, 909, 19,862, 238, 19,624” are replaced by the correct numbers “32,709, 28,591, 28,105, 312, 174, 4118, 115, 4003”. The plots from the results of restricted cubic spline models in Supplementary Fig. 2 and 3 were substituted with the corrected ones.

The original versions of Tables 1 and 2, and those of Fig. 1, 2, 3, 4, and 5 have now been replaced with corrected versions.

The previous version of Table 1 was:

Table 1. Baseline characteristics of 19,624 participants by MVPA.

**Table:** 

Characteristics	Total	Device-measured MVPA, minutes/week			
		<275	275–449	450–624	≥625
Total, n	19,624	4148	5517	4936	5023
Age, year, mean (SD)	62.3 (7.8)	66.0 (6.7)	63.2 (7.5)	61.3 (7.7)	59.0 (7.5)
Sex, male, n (%)	8713 (44.4)	2063 (49.7)	2492 (45.2)	2112 (42.8)	2046 (40.7)
Ethnicity, White, n (%)	18,761 (95.6)	4006 (96.6)	5306 (96.2)	4696 (95.1)	4753 (94.6)
Education, college or university, n (%)	8057 (41.1)	1424 (34.3)	2197 (39.8)	2173 (44.0)	2263 (45.1)
BMI, kg/m^2^, mean (SD)	27.3 (5.0)	29.9 (5.9)	27.8 (5.0)	26.6 (4.3)	25.5 (3.9)
Waist circumference, cm, mean (SD)	90.1 (14.2)	97.7 (15.3)	91.6 (13.9)	87.9 (12.7)	84.5 (12.0)
**Smoking status, n (%)**					
Never	10,910 (55.6)	2009 (48.4)	3035 (55.0)	2857 (57.9)	3009 (59.9)
Former	7315 (37.3)	1714 (41.3)	2091 (37.9)	1782 (36.1)	1728 (34.4)
Current	1399 (7.1)	425 (10.2)	391 (7.1)	297 (6.0)	286 (5.7)
Diet score, mean (SD)	4.0 (2.0)	3.9 (2.1)	4.0 (2.0)	4.1 (2.0)	4.1 (2.0)
Sleep score, mean (SD)	3.1 (1.0)	2.8 (1.0)	3.0 (1.0)	3.1 (1.0)	3.2 (1.0)
Alcohol intake, g/day, mean (SD)	17.89 (19.91)	16.7 (21.8)	17.6 (19.4)	18.8 (19.7)	18.4 (18.9)
LPA, minutes/week, mean (SD)	2036.1 (456.2)	1702.4 (423.9)	1994.3 (405.2)	2125.0 (402.2)	2270.1 (411.1)
MPA, minutes/week, mean (SD)	460.3 (238.0)	179.0 (61.4)	347.1 (48.2)	502.8 (55.6)	775.0 (180.1)
VPA, minutes/week, mean (SD)	28.9 (39.9)	5.1 (7.9)	16.1 (16.6)	30.2 (29.4)	61.3 (57.4)
**Season of wear, n (%)**					
Spring	4270 (21.8)	834 (20.1)	1140 (20.7)	1132 (22.9)	1164 (23.2)
Summer	5320 (27.1)	1080 (26.0)	1493 (27.1)	1326 (26.9)	1421 (28.3)
Autumn	5726 (29.2)	1205 (29.1)	1639 (29.7)	1425 (28.9)	1457 (29.0)
Winter	4308 (22.0)	1029 (24.8)	1245 (22.6)	1053 (21.3)	981 (19.5)
Wear duration, day, mean (SD)	6.7 (0.7)	6.7 (0.7)	6.7 (0.6)	6.7 (0.6)	6.7 (0.7)
Diabetes duration, year, mean (SD)	5.8 (2.1)	6.2 (2.8)	5.9 (2.1)	5.7 (1.7)	5.7 (1.5)
HbA1c, mean (SD)	39.0 (10.5)	43.5 (12.8)	39.2 (10.6)	37.3 (8.6)	36.3 (7.9)
Insulin medication use, n (%)	564 (2.9)	227 (5.5)	161 (2.9)	90 (1.8)	86 (1.7)
**Self-rated health, n (%)**					
Excellent	3730 (19.0)	430 (10.4)	886 (16.1)	1096 (22.2)	1318 (26.2)
Good	11,393 (58.1)	2101 (50.7)	3287 (59.6)	2965 (60.1)	3040 (60.5)
Fair	3690 (18.8)	1197 (28.9)	1119 (20.3)	772 (15.6)	602 (12.0)
Poor	811 (4.1)	420 (10.1)	225 (4.1)	103 (2.1)	63 (1.3)
History of cancer or CVD, n (%)	3294 (16.8)	1187 (28.6)	964 (17.5)	673 (13.6)	470 (9.4)
History of hypertension, n (%)	5,312 (27.1)	1859 (44.8)	1604 (29.1)	1055 (21.4)	794 (15.8)
Long-standing illness, disability or infirmity, n (%)	6854 (34.9)	2261 (54.5)	2,018 (36.6)	1,418 (28.7)	1157 (23.0)
Illness, injury, bereavement, or stress in last 2 years, n (%)	8379 (42.7)	1937 (46.7)	2368 (42.9)	2036 (41.2)	2038 (40.6)

*BMI* indicates body mass index, *CVD* cardiovascular disease, *LPA* light-intensity physical activity, *MPA* moderate-intensity physical activity, *VPA* vigorous-intensity physical activity, *MVPA* moderate-to-vigorous-intensity physical activity, *SD* standard deviation.

The correct version appears as:

Table 1. Baseline characteristics of 4003 participants by MVPA

**Table:** 

Characteristics	Total	Device-measured MVPA, min/week			
		<275	275–449	450–624	≥625
Total, n	4003	1665	1197	684	457
Age, year, mean (SD)	64.9 (6.9)	66.8 (6.1)	64.5 (6.9)	63.3 (7.2)	61.2 (7.3)
Sex, male, n (%)	2523 (63.0)	1044 (62.7)	769 (64.2)	427 (62.4)	283 (61.9)
Ethnicity, White, n (%)	3740 (93.4)	1587 (95.3)	1129 (94.3)	609 (89.0)	415 (90.8)
Education, college or university, n (%)	1332 (33.3)	492 (29.5)	418 (34.9)	255 (37.3)	167 (36.5)
BMI, kg/m^2^, mean (SD)	31.1 (5.8)	32.5 (6.1)	31.0 (5.7)	29.7 (4.8)	28.1 (4.8)
Waist circumference, cm, mean (SD)	102.0 (14.4)	105.8 (14.3)	101.6 (13.7)	98.7 (13.0)	93.7 (13.6)
Smoking status, n (%)					
Never	1760 (44.0)	650 (39.0)	560 (46.8)	327 (47.8)	223 (48.8)
Former	1905 (47.6)	842 (50.6)	541 (45.2)	311 (45.5)	211 (46.2)
Current	338 (8.4)	173 (10.4)	96 (8.0)	46 (6.7)	23 (5.0)
Diet score, mean (SD)	3.7 (1.9)	3.6 (2.0)	3.7 (1.8)	3.8 (1.9)	4.1 (2.0)
Sleep score, mean (SD)	2.8 (1.0)	2.7 (1.0)	2.9 (1.0)	2.9 (1.0)	3.0 (1.1)
Alcohol intake, g/day, mean (SD)	16.8 (21.6)	14.9 (20.8)	17.5 (22.3)	18.3 (21.8)	20.0 (21.7)
LPA, min/week, mean (SD)	1901.3 (478.0)	1648.1 (437.4)	1990.2 (408.8)	2125.5 (418.5)	2255.7 (387.6)
MPA, min/week, mean (SD)	340.8 (206.5)	163.8 (64.1)	340.3 (47.5)	498.2 (55.4)	751.2 (169.0)
VPA, min/week, mean (SD)	16.6 (27.5)	4.3 (7.9)	15.0 (15.1)	27.3 (28.7)	49.7 (52.3)
Season of wear, n (%)					
Spring	848 (21.2)	336 (20.2)	247 (20.6)	164 (24.0)	101 (22.1)
Summer	1108 (27.7)	434 (26.1)	338 (28.2)	205 (30.0)	131 (28.7)
Autumn	1152 (28.8)	491 (29.5)	355 (29.7)	168 (24.6)	138 (30.2)
Winter	895 (22.4)	404 (24.3)	257 (21.5)	147 (21.5)	87 (19.0)
Wear duration, day, mean (SD)	6.7 (0.7)	6.7 (0.7)	6.7 (0.6)	6.7 (0.7)	6.6 (0.7)
Diabetes duration, year, mean (SD)	11.2 (9.4)	11.5 (9.1)	10.8 (8.8)	11.0 (10.3)	11.8 (10.6)
HbA1c, mean (SD)	50.9 (13.0)	51.7 (13.2)	50.4 (13.2)	49.9 (11.7)	50.8 (13.6)
Insulin medication use, n (%)	601 (15.1)	235 (14.1)	171 (14.3)	98 (14.4)	97 (21.3)
Self-rated health, n (%)					
Excellent	200 (5.0)	61 (3.7)	52 (4.3)	52 (7.6)	35 (7.7)
Good	1851 (46.2)	634 (38.1)	599 (50.0)	357 (52.2)	261 (57.1)
Fair	1476 (36.9)	688 (41.3)	421 (35.2)	233 (34.1)	134 (29.3)
Poor	476 (11.9)	282 (16.9)	125 (10.4)	42 (6.1)	27 (5.9)
History of cancer or CVD, n (%)	1201 (30.0)	628 (37.7)	338 (28.2)	168 (24.6)	67 (14.7)
History of hypertension, n (%)	2308 (57.7)	1128 (67.7)	647 (54.1)	321 (46.9)	212 (46.4)
Long-standing illness, disability or infirmity, n (%)	2980 (74.4)	1335 (80.2)	864 (72.2)	482 (70.5)	299 (65.4)
Illness, injury, bereavement, or stress in last 2 years, n (%)	1962 (49.0)	851 (51.1)	585 (48.9)	326 (47.7)	200 (43.8)

*BMI* body mass index, *CVD* cardiovascular disease, *LPA* light-intensity physical activity, *MPA* moderate-intensity physical activity, *VPA* vigorous-intensity physical activity, *MVPA* moderate-to-vigorous-intensity physical activity, *SD* standard deviation.

The previous version of Table 2 was:

Table 2. Hazard ratios (95% CI) for all-cause mortality according to physical activity among individuals with type 2 diabetes

**Table:** 

Exposures	No. of cases	Incidence rate per 1000 person-year	Hazard ratio (95% CI)^a^		
			Model 1	Model 2	Model 3
**LPA (minutes/week)**					
<1750	336	9.58	1 (Ref)	1 (Ref)	1 (Ref)
1750–2099	229	5.62	0.66 (0.56,0.78)	0.68 (0.58,0.81)	0.77 (0.65,0.92)
2100–2449	149	4.27	0.53 (0.44,0.65)	0.55 (0.45,0.67)	0.65 (0.53,0.79)
≥2450	96	4.02	0.54 (0.43,0.68)	0.56 (0.44,0.71)	0.68 (0.54,0.87)
p value for trend			<0.001	<0.001	<0.001
**MPA (minutes/week)**					
<150	182	23.56	1 (Ref)	1 (Ref)	1 (Ref)
150–299	255	9.40	0.47 (0.39,0.57)	0.50 (0.41,0.60)	0.57 (0.47,0.70)
300–449	190	5.20	0.31 (0.26,0.39)	0.34 (0.27,0.42)	0.42 (0.34,0.53)
≥450	183	2.90	0.22 (0.18,0.27)	0.24 (0.19,0.30)	0.32 (0.25,0.40)
p value for trend			<0.001	<0.001	<0.001
**VPA (minutes/week)**					
0	322	13.36	1 (Ref)	1 (Ref)	1 (Ref)
1–14	229	5.90	0.52 (0.44,0.62)	0.54 (0.46,0.64)	0.61 (0.52,0.73)
15–29	172	4.61	0.45 (0.37,0.54)	0.47 (0.39,0.57)	0.57 (0.47,0.70)
≥30	87	2.53	0.30 (0.24,0.38)	0.33 (0.26,0.42)	0.42 (0.33,0.55)
p value for trend			<0.001	<0.001	<0.001
**MVPA (minutes/week)**					
<275	394	14.27	1 (Ref)	1 (Ref)	1 (Ref)
275–449	202	5.33	0.46 (0.39,0.55)	0.49 (0.41,0.58)	0.55 (0.47,0.66)
450–624	134	3.92	0.40 (0.33,0.49)	0.42 (0.35,0.52)	0.51 (0.42,0.63)
≥625	80	2.29	0.29 (0.22,0.37)	0.31 (0.24,0.40)	0.39 (0.30,0.50)
p value for trend			<0.001	<0.001	<0.001

*HR* indicates hazard ratio, *CI* confidence interval, *LPA* light-intensity physical activity, *MPA* moderate-intensity physical activity, *VPA* vigorous-intensity physical activity, *MVPA* moderate-to-vigorous-intensity physical activity.

^a^Hazard ratios (95% CI) were calculated in Cox proportional hazards model: model 1, adjusted for age (years), sex (male or female), ethnicity (white or others), education (college/university or others), season at the time of accelerometry recording (spring, summer, autumn, or winter), and accelerometer wear duration (days); model 2, further adjusted for smoking status (never, former, or current), alcohol intake (g/day), diet score (0 to 7), and sleep score (0 to 5) based on model 1; model 3, further adjusted for body mass index (kg/m^2^), waist circumference (cm), self-rated health (excellent, good, fair, or poor), long-standing illness, disability or infirmity (yes or no), illness, injury, bereavement, or stress in last 2 years (yes or no), history of cancer or cardiovascular disease (yes or no), history of hypertension (yes or no), and diabetes duration (years) based on model 2. Wald tests were used to obtain the two-sided p-value.

The correct version appears as:

Table 2. Hazard ratios (95% CI) for all-cause mortality according to physical activity among individuals with type 2 diabetes

**Table:** 

Exposures	No. of cases	Incidence rate per 1000 person-year	Hazard ratio (95% CI)^a^		
			Model 1	Model 2	Model 3
LPA (min/week)					
<1750	167	16.66	1.00 (Ref)	1.00 (Ref)	1.00 (Ref)
1750–2099	102	12.93	0.82 (0.64,1.05)	0.83 (0.65,1.06)	0.90 (0.70,1.16)
2100–2449	45	8.03	0.53 (0.38,0.74)	0.55 (0.40,0.77)	0.60 (0.43,0.84)
≥2450	25	7.48	0.53 (0.35,0.82)	0.55 (0.36,0.84)	0.60 (0.39,0.93)
p-value for trend			<0.001	<0.001	=0.001
MPA (min/week)					
<150	119	28.46	1.00 (Ref)	1.00 (Ref)	1.00 (Ref)
150–299	129	15.03	0.58 (0.45,0.75)	0.59 (0.46,0.76)	0.61 (0.47,0.79)
300–449	61	8.60	0.36 (0.26,0.49)	0.38 (0.27,0.52)	0.41 (0.29,0.56)
≥450	30	4.28	0.20 (0.13,0.31)	0.21 (0.14,0.32)	0.24 (0.15,0.36)
p-value for trend			<0.001	<0.001	<0.001
VPA (min/week)					
0	183	20.91	1.00 (Ref)	1.00 (Ref)	1.00 (Ref)
1-14	89	9.97	0.53 (0.41,0.68)	0.54 (0.42,0.70)	0.58 (0.44,0.75)
15-29	48	8.30	0.46 (0.33,0.64)	0.48 (0.34,0.66)	0.52 (0.37,0.73)
≥30	19	5.57	0.35 (0.22,0.57)	0.37 (0.23,0.61)	0.43 (0.26,0.70)
p-value for trend			<0.001	<0.001	<0.001
MVPA (min/week)					
<275	228	21.00	1.00 (Ref)	1.00 (Ref)	1.00 (Ref)
275–449	73	9.01	0.48 (0.37,0.63)	0.51 (0.39,0.66)	0.54 (0.41,0.71)
450–624	26	5.50	0.32 (0.21,0.48)	0.33 (0.22,0.49)	0.36 (0.24,0.55)
≥625	12	3.78	0.25 (0.14,0.45)	0.27 (0.15,0.49)	0.30 (0.16,0.54)
p-value for trend			<0.001	<0.001	<0.001

*HR* hazard ratio, *CI* confidence interval, *LPA* light-intensity physical activity, *MPA* moderate-intensity physical activity, *VPA* vigorous-intensity physical activity, *MVPA* moderate-to-vigorous-intensity physical activity.

^a^Hazard ratios (95% CI) were calculated in Cox proportional hazards model: model 1, adjusted for age (years), sex (male or female), ethnicity (white or others), education (college/university or others), season at the time of accelerometry recording (spring, summer, autumn, or winter), and accelerometer wear duration (days); model 2, further adjusted for smoking status (never, former, or current), alcohol intake (g/day), diet score (0 to 7), and sleep score (0 to 5) based on model 1; model 3, further adjusted for body mass index (kg/m^2^), waist circumference (cm), self-rated health (excellent, good, fair, or poor), long-standing illness, disability or infirmity (yes or no), illness, injury, bereavement, or stress in last 2 years (yes or no), history of cancer or cardiovascular disease (yes or no), history of hypertension (yes or no), and diabetes duration (years) based on model 2. Wald tests were used to obtain the two-sided p-value.

The previous version of Figure 1 was:
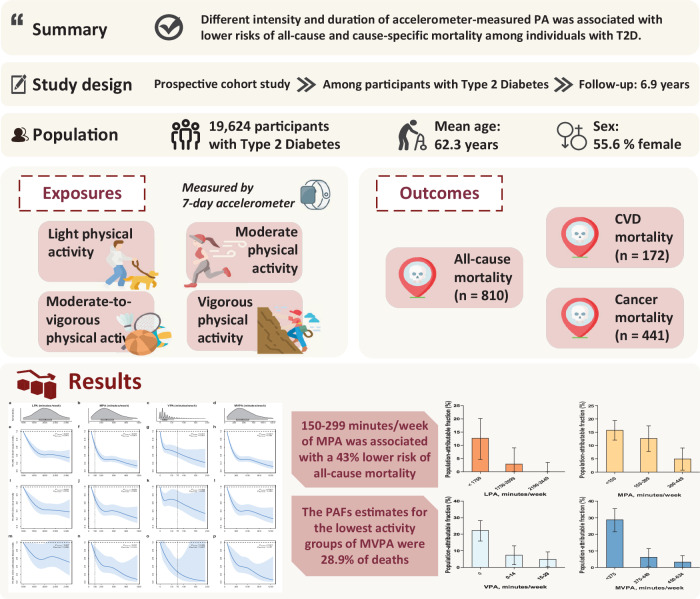


The correct version appears as:
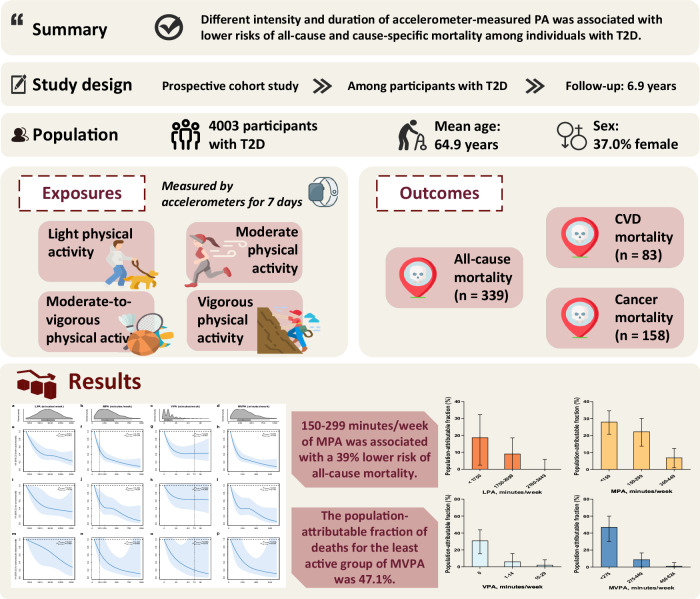


The previous version of Figure 2 was:
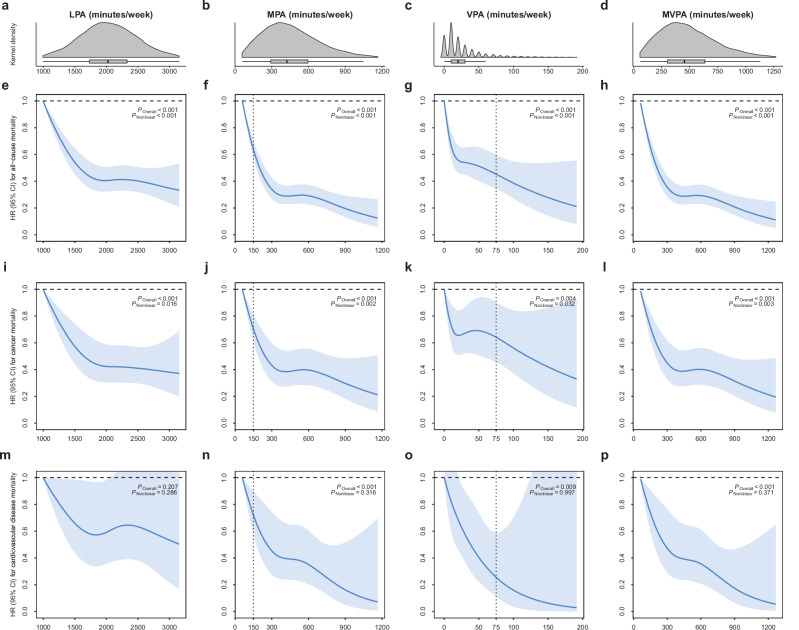


The correct version appears as:
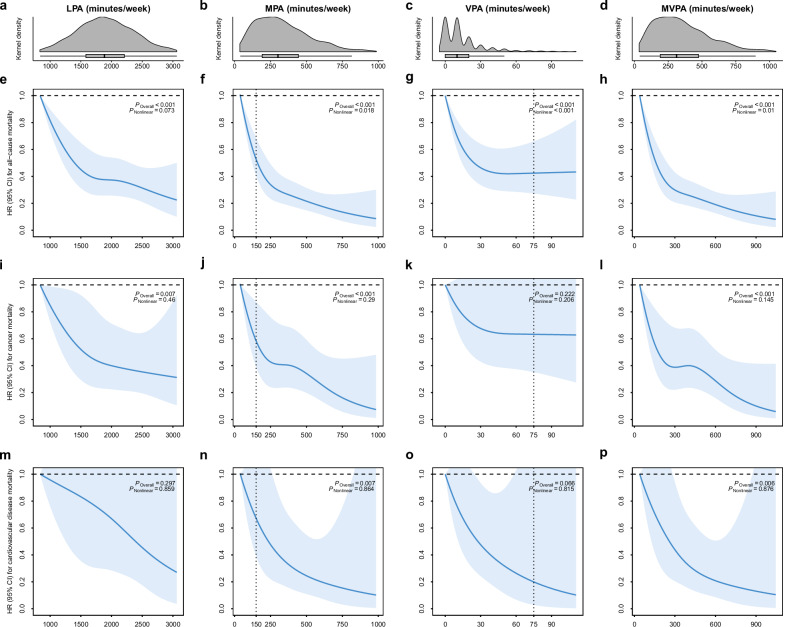


The previous version of Figure 3 was:
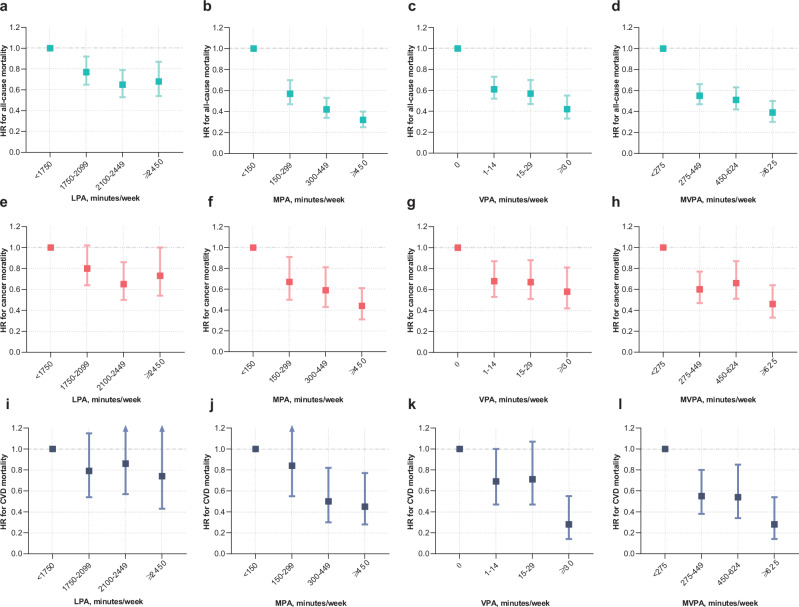


The correct version appears as:
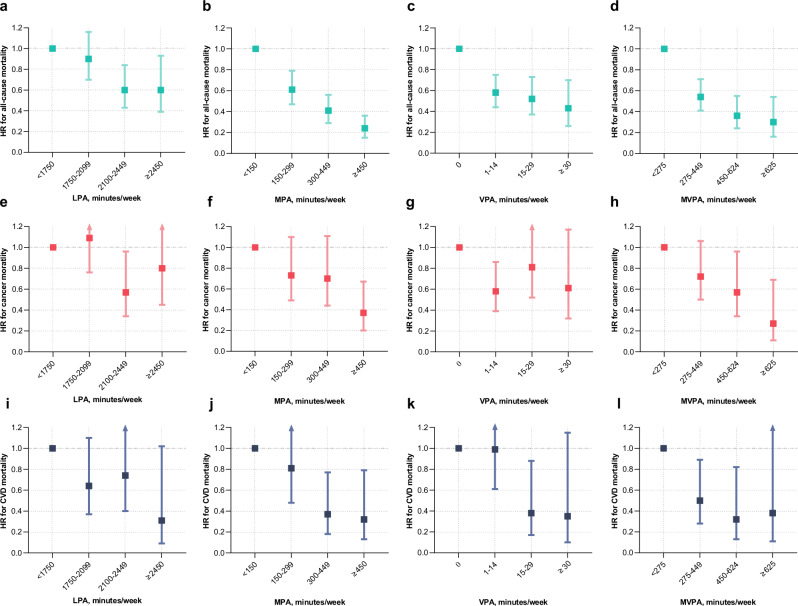


The previous version of Figure 4 was:
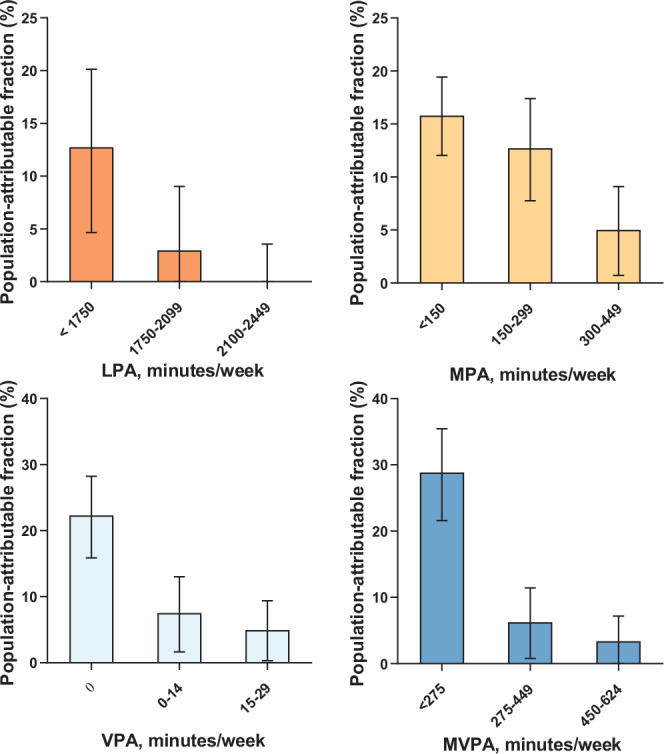


The correct version appears as:
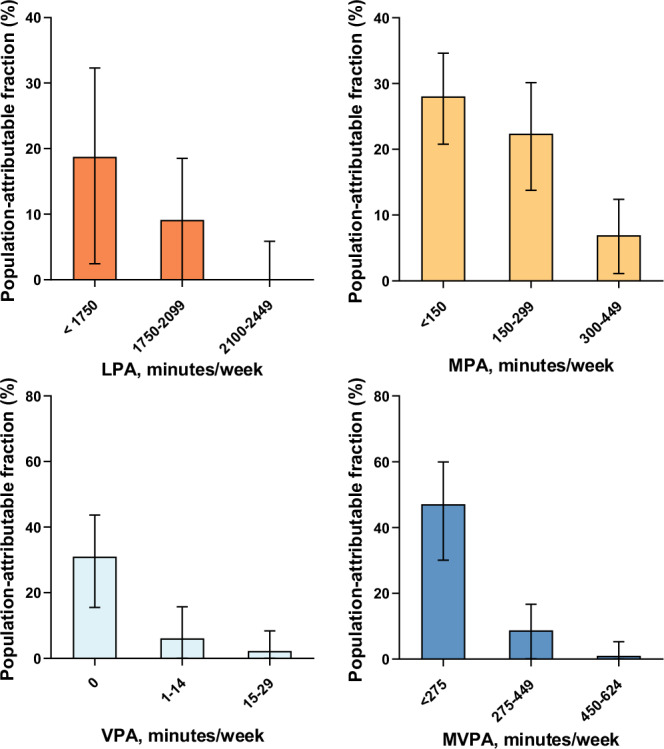


The previous version of Figure 5 was:
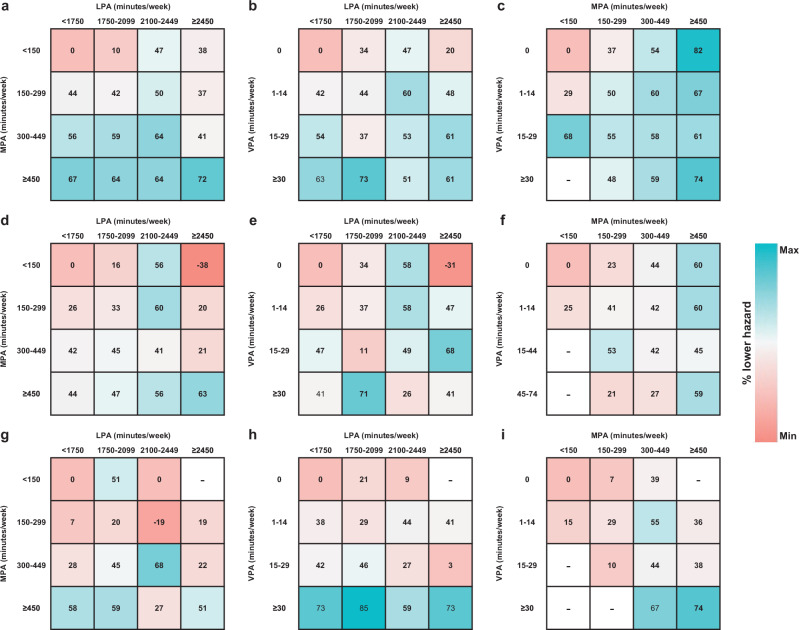


The correct version appears as:
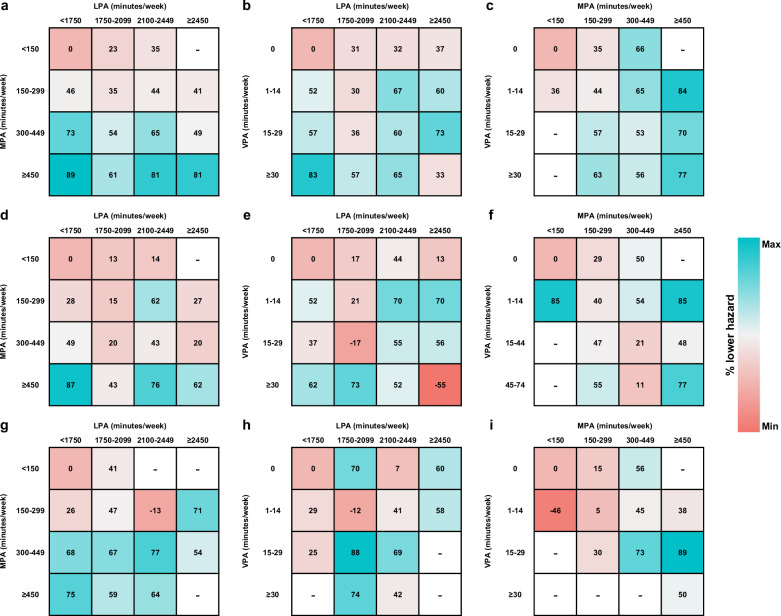


## Supplementary information

Updated Supplementary information

## Source data

Updated Source data.

These errors have been corrected in the HTML and PDF versions of the article. The HTML has been updated to include a corrected version of the Supplementary information. The Source Data File has been updated accordingly.

