# Supplementary material for: Accelerometer-derived physical activity and mortality in individuals with type 2 diabetes
Source: Nat Commun. 2024 Jun 17;15:5164. doi: 10.1038/s41467-024-49542-0 (PMC11183112; doi:10.1038/s41467-024-49542-0)
Supplement: Supplementary file 1 — Supplementary Information [file 41467_2024_49542_MOESM1_ESM.doc]

**Accelerometer-derived physical activity and mortality in individuals with type 2 diabetes**

Zhi Cao^1,2,6^, Jiahao Min^1,6^, Han Chen^1^, Yabing Hou^3^, Hongxi Yang^4^, Keyi Si^5,7^, Chenjie Xu^1,7^

^1^ School of Public Health, Hangzhou Normal University, Hangzhou, China.

^2^ School of Public Health, Zhejiang University School of Medicine, Hangzhou, China.

^3^ Yanjing Medical College, Capital Medical University, Beijing, China.

^4^ School of Basic Medical Sciences, Tianjin Medical University, Tianjin, China.

^5^ School of Public Health, Shanghai Jiao Tong University School of Medicine, Shanghai, China.

^6^ These authors contributed equally: Zhi Cao, Jiahao Min.

^7^ These authors jointly supervised this work: Keyi Si, Chenjie Xu.

e-mail: [sikeyi0219@163.com](mailto:sikeyi0219@163.com); xuchenjie@hznu.edu.cn.

***Supplementary material***

eMethods. Supplementary methods.

Supplementary Table 1. Characteristics of participants with type 2 diabetes included in or excluded from the current study.

Supplementary Table 2. Baseline characteristics of 4003 participants by LPA in the UK Biobank.

Supplementary Table 3. Baseline characteristics of 4003 participants by MPA in the UK Biobank.

Supplementary Table 4. Baseline characteristics of 4003 participants by VPA in the UK Biobank.

Supplementary Table 5. Population-attributable fractions (PAFs) for all-cause mortality associated with physical activity.

Supplementary Table 6. Hazard ratios (95% CI) for cancer mortality according to physical activity among individuals with type 2 diabetes.

Supplementary Table 7. Hazard ratios (95% CI) for cardiovascular-disease mortality according to physical activity among individuals with type 2 diabetes.

Supplementary Table 8. Joint association of LPA and MPA with all-cause and cause-specific mortality for the risk matrix.

Supplementary Table 9. Joint association of LPA and VPA with all-cause and cause-specific mortality for the risk matrix.

Supplementary Table 10. Joint association of MPA and VPA with all-cause and cause-specific mortality for the risk matrix.

Supplementary Table 11. Association between LPA and all-cause mortality among participants with type 2 diabetes in subgroups *.

Supplementary Table 12. Association between MPA and all-cause mortality among participants with type 2 diabetes in subgroups *.

Supplementary Table 13. Association between VPA and all-cause mortality among participants with type 2 diabetes in subgroups *.

Supplementary Table 14. Association between MVPA and all-cause mortality among participants with type 2 diabetes in subgroups *.

Supplementary Table 15. Association of physical activity with all-cause and cause-specific mortality risk, excluding patients with poor self-rated health (n=3527).

Supplementary Table 16. Association between physical activity and all-cause mortality risk stratified by diabetes severity status (n = 3766) *.

Supplementary Table 17. Association of physical activity with all-cause mortality risk after adjusting for diabetes severity factors (n = 3766).

Supplementary Table 18. Association between physical activity and all-cause and cause-specific mortality risk, additionally adjusted for diabetes related disease.

Supplementary Table 19. Association between physical activity and all-cause and cause-specific mortality risk, mutually adjusted for different PA intensities.

Supplementary Table 20. Association of physical activity with all-cause and cause-specific mortality risk using multiple imputations with chained equations (n = 4118).

Supplementary Table 21. Association of physical activity with cancer and cardiovascular disease mortality risk using Fine & Gray models for competing risk (n = 4003).

Supplementary Table 22. Association between physical activity and all-cause and cause-specific mortality risk converting the duration data into MET-minutes/week.

Supplementary Table 23. Association between physical activity and all-cause and cause-specific mortality risk grouping participants into two groups.

Supplementary Table 24. Association between physical activity and risk of mortality from cancer and CVD among participants without the corresponding disease at baseline.

Supplementary Table 25. Association of physical activity with all-cause and cause-specific mortality risk, excluding deaths that occurred within the first 2 or 4 years of follow-up.

Supplementary Table 26. Association of physical activity with T2D/Obesity-related or -independent cancers mortality risk (n = 4003).

Supplementary Table 27. Proportional hazard test for the main model.

Supplementary Fig. 1. Flowchart of participants included in the analysis.

Supplementary Fig. 2. Dose-response association between physical activity and all-cause and cause-specific mortality, mutually adjusted for PA intensities.

Supplementary Fig. 3. Dose-response association between total physical activity and all-cause and cause-specific mortality using the MET-minutes/week as the unit.

Supplementary Fig. 4. The directed acyclic graph to guide covariate selection.

**eMethods. Supplementary methods.**

*Multiple imputation*

We used multiple imputations by chained equations to impute missing values for the following covariates: ethnicity, education, BMI, waist, alcohol intake, smoking status, diet scores, diabetes duration, self-rated health status, long-standing illness, disability or infirmity, illness, injury, bereavement, stress in last 2 years. The imputations model included all of covariates as predictors. We performed 10 imputations and pooled all results across the imputed datasets using Rubin’s rules to obtain final effect estimates.

*Missing proportion of covariates needed to impute*

Ethnicity (0.36%), education (1.29%), BMI (0.53%), waist (0.27%), alcohol intake (missing proportion: 0.01%), smoking status (0.39%), diet scores (4.18%), diabetes duration (0.01%), self-rated health status (0.46%), long-standing illness, disability or infirmity (1.80%), illness, injury, bereavement, stress in last 2 years (0.97%).

**Supplementary Table 1. Characteristics of participants with type 2 diabetes included in or excluded from the current study.**

| **Characteristics** | **Excluded*** | **Included** |
| --- | --- | --- |
| **Total, n** | 28,706 | 4003 |
| **Age, year, mean (SD)** | 59.6 (7.2) | 59.2 (6.9) |
| **Sex, male, n (%)** | 17,614 (61.4) | 2523 (63.0) |
| **Ethnicity, white, n (%)** | 24,525 (86.4) | 3740 (93.4) |
| **Education, college or university, n (%)** | 6062 (21.9) | 1332 (33.5) |
| **BMI, kg/m^2^, mean (SD)** | 31.5 (5.9) | 31.1 (5.8) |
| **Waist, cm, mean (SD)** | 103.1 (14.3) | 102.0 (14.4) |
| **Smoking status, n (%)** |  |  |
| Never | 12,884 (45.5) | 1760 (44.0) |
| Former | 11,934 (42.1) | 1905 (47.6) |
| Current | 3524 (12.4) | 338 (8.4) |
| **Diet score, mean (SD)** | 3.5 (1.5) | 3.5 (1.5) |
| **Sleep score, mean (SD)** | 2.7 (1.1) | 2.8 (1.0) |
| **Alcohol intake, g/day, mean (SD)** | 14.4 (21.4) | 16.8 (21.6) |
| **HbA1c, mean (SD)** | 52.9 (14.9) | 50.9 (13.0) |
| **Insulin medication use, n (%)** | 5010 (17.9) | 601 (15.1) |
| **Self-rated health, n (%)** |  |  |
| Excellent | 929 (3.3) | 200 (5.0) |
| Good | 10,796 (38.2) | 1851 (46.2) |
| Fair | 11,697 (41.4) | 1476 (36.9) |
| poor | 4820 (17.1) | 476 (11.9) |
| **History of cancer or CVD, n (%)** | 9776 (34.1) | 1201 (30.0) |
| **History of hypertension, n (%)** | 17,636 (61.4) | 2308 (57.7) |
| **Long-standing illness, disability or infirmity, n (%)** | 21,468 (77.1) | 2980 (75.7) |
| **Illness, injury, bereavement, stress in last 2 years, n (%)** | 14,679 (52.7) | 1962 (49.0) |

* Participants with missing information or poor-quality information on wear time, quality of wear, daylight saving crossover, smoking status, alcohol intake, BMI, ethnicity, waist circumference, self-rated health at baseline, diabetes duration, and illness, injury, bereavement, or stress in last 2 years were excluded.

The age information in this table is data from the baseline survey (2006-2010).

BMI indicates body mass index; CVD, cardiovascular disease; SD, standard deviation.

**Supplementary Table 2. Baseline characteristics of 4003 participants by LPA in the UK Biobank.**

| **Characteristics** | **Total** | **Device-measured LPA, minutes/week** | | | |
| --- | --- | --- | --- | --- | --- |
|  |  | **<1750** | **1750-2099** | **2100-2449** | **≥2450** |
| **Total, n** | 4003 | 1515 | 1173 | 822 | 493 |
| **Age, year, mean (SD)** | 64.9 (6.9) | 65.5 (6.8) | 64.8 (6.8) | 64.4 (7.0) | 63.9 (7.2) |
| **Sex, male, n (%)** | 2523 (63.0) | 1054 (69.6) | 741 (63.2) | 480 (58.4) | 248 (50.3) |
| **Ethnicity, white, n (%)** | 3740 (93.4) | 1431 (94.5) | 1115 (95.1) | 759 (92.3) | 435 (88.2) |
| **Education, college or university, n (%)** | 1332 (33.3) | 499 (32.9) | 392 (33.4) | 274 (33.3) | 167 (33.9) |
| **BMI, kg/m^2^, mean (SD)** | 31.1 (5.8) | 32.1 (6.3) | 30.8 (5.4) | 30.4 (5.4) | 29.6 (5.2) |
| **Waist circumference, cm, mean (SD)** | 102.0 (14.4) | 105.2 (14.6) | 101.4 (13.5) | 99.8 (14.3) | 97.1 (13.8) |
| **Smoking status, n (%)** |  |  |  |  |  |
| Never | 1760 (44.0) | 635 (41.9) | 485 (41.3) | 404 (49.1) | 236 (47.9) |
| Former | 1905 (47.6) | 722 (47.7) | 604 (51.5) | 360 (43.8) | 219 (44.4) |
| Current | 338 (8.4) | 158 (10.4) | 84 (7.2) | 58 (7.1) | 38 (7.7) |
| **Diet score, mean (SD)** | 3.7 (1.9) | 3.6 (2.0) | 3.7 (1.8) | 3.8 (1.9) | 4.1 (2.0) |
| **Sleep score, mean (SD)** | 2.8 (1.0) | 2.7 (1.0) | 2.9 (1.0) | 2.9 (1.0) | 2.9 (1.0) |
| **Alcohol intake, g/day, mean (SD)** | 16.8 (21.6) | 15.7 (20.7) | 18.4 (22.1) | 16.8 (21.6) | 16.7 (22.8) |
| **MPA, minutes/week, mean (SD)** | 340.8 (206.5) | 230.0 (154.9) | 351.1 (181.2) | 434.8 (204.5) | 499.7 (216.6) |
| **VPA, minutes/week, mean (SD)** | 16.6 (27.5) | 10.9 (22.7) | 18.0 (28.9) | 21.1 (26.9) | 23.4 (34.3) |
| **MVPA, minutes/week, mean (SD)** | 357.5 (220.7) | 241.0 (166.4) | 369.3 (196.0) | 456.1 (219.2) | 523.2 (230.9) |
| **Season of wear, n (%)** |  |  |  |  |  |
| Spring | 848 (21.2) | 319 (21.1) | 254 (21.7) | 167 (20.3) | 108 (21.9) |
| Summer | 1108 (27.7) | 373 (24.6) | 314 (26.8) | 253 (30.8) | 168 (34.1) |
| Autumn | 1152 (28.8) | 440 (29.0) | 346 (29.5) | 240 (29.2) | 126 (25.6) |
| Winter | 895 (22.4) | 383 (25.3) | 259 (22.1) | 162 (19.7) | 91 (18.5) |
| **Wear duration, day, mean (SD)** | 6.7 (0.7) | 6.7 (0.7) | 6.7 (0.6) | 6.7 (0.7) | 6.6 (0.7) |
| **Diabetes duration, year, mean (SD)** | 11.2 (9.4) | 11.3 (9.1) | 11.2 (9.1) | 10.9 (9.6) | 11.7 (10.7) |
| **HbA1c, mean (SD)** | 50.9 (13.0) | 50.9 (12.5) | 51.4 (13.8) | 50.5 (13.1) | 50.6 (12.3) |
| **Insulin medication use, n (%)** | 601 (15.1) | 207 (13.7) | 164 (14.0) | 134 (16.4) | 96 (19.5) |
| **Self-rated health, n (%)** |  |  |  |  |  |
| Excellent | 200 (5.0) | 64 (4.2) | 56 (4.8) | 51 (6.2) | 29 (5.9) |
| Good | 1851 (46.2) | 592 (39.1) | 604 (51.5) | 398 (48.4) | 257 (52.1) |
| Fair | 1476 (36.9) | 612 (40.4) | 397 (33.8) | 305 (37.1) | 162 (32.9) |
| poor | 476 (11.9) | 247 (16.3) | 116 (9.9) | 68 (8.3) | 45 (9.1) |
| **History of cancer or CVD, n (%)** | 1201 (30.0) | 532 (35.1) | 346 (29.5) | 219 (26.6) | 104 (21.1) |
| **History of hypertension, n (%)** | 2308 (57.7) | 960 (63.4) | 663 (56.5) | 443 (53.9) | 242 (49.1) |
| **Long-standing illness, disability or infirmity, n (%)** | 2980 (74.4) | 1189 (78.5) | 869 (74.1) | 580 (70.6) | 342 (69.4) |
| **Illness, injury, bereavement, stress in last 2 years, n (%)** | 1962 (49.0) | 767 (50.6) | 552 (47.1) | 392 (47.7) | 251 (50.9) |

BMI indicates body mass index; CVD, cardiovascular disease; LPA, light-intensity physical activity; MPA, moderate-intensity physical activity; VPA, vigorous-intensity physical activity; MVPA, moderate-to-vigorous-intensity physical activity; SD, standard deviation.

**Supplementary Table 3. Baseline characteristics of 4003 participants by MPA in the UK Biobank.**

| **Characteristics** | **Total** | **Device-measured MPA, minutes/week** | | | |
| --- | --- | --- | --- | --- | --- |
|  |  | **<150** | **150-299** | **300-449** | **≥450** |
| **Total, n** | 4003 | 659 | 1288 | 1045 | 1011 |
| **Age, year, mean (SD)** | 64.9 (6.9) | 67.7 (5.8) | 65.8 (6.3) | 64.4 (7.0) | 62.3 (7.3) |
| **Sex, male, n (%)** | 2523 (63.0) | 413 (62.7) | 821 (63.7) | 664 (63.5) | 625 (61.8) |
| **Ethnicity, white, n (%)** | 3740 (93.4) | 635 (96.4) | 1218 (94.6) | 982 (94.0) | 905 (89.5) |
| **Education, college or university, n (%)** | 1332 (33.3) | 186 (28.2) | 397 (30.8) | 374 (35.8) | 375 (37.1) |
| **BMI, kg/m^2^, mean (SD)** | 31.1 (5.8) | 33.5 (6.2) | 31.8 (5.9) | 30.6 (5.6) | 29.0 (4.8) |
| **Waist circumference, cm, mean (SD)** | 102.0 (14.4) | 108.4 (14.1) | 104.0 (14.1) | 100.8 (13.8) | 96.4 (13.2) |
| **Smoking status, n (%)** |  |  |  |  |  |
| Never | 1760 (44.0) | 249 (37.8) | 528 (41.0) | 493 (47.2) | 490 (48.5) |
| Former | 1905 (47.6) | 326 (49.5) | 648 (50.3) | 472 (45.2) | 459 (45.4) |
| Current | 338 (8.4) | 84 (12.7) | 112 (8.7) | 80 (7.7) | 62 (6.1) |
| **Diet score, mean (SD)** | 3.7 (1.9) | 3.7 (2.1) | 3.6 (1.8) | 3.7 (1.8) | 3.9 (1.9) |
| **Sleep score, mean (SD)** | 2.8 (1.0) | 2.7 (1.0) | 2.8 (1.0) | 2.9 (1.0) | 3.0 (1.0) |
| **Alcohol intake, g/day, mean (SD)** | 16.8 (21.6) | 13.4 (21.2) | 16.6 (21.4) | 17.5 (21.8) | 18.8 (21.6) |
| **LPA, minutes/week, mean (SD)** | 1901.3 (478.0) | 1422.3 (400.7) | 1827.1 (401.1) | 2003.6 (408.8) | 2202.5 (405.7) |
| **VPA, minutes/week, mean (SD)** | 16.6 (27.5) | 1.7 (6.4) | 8.9 (18.9) | 18.7 (27.9) | 34.0 (34.3) |
| **MVPA, minutes/week, mean (SD)** | 357.5 (220.7) | 99.2 (35.8) | 232.2 (50.3) | 386.0 (54.3) | 656.0 (179.5) |
| **Season of wear, n (%)** |  |  |  |  |  |
| Spring | 848 (21.2) | 141 (21.4) | 252 (19.6) | 220 (21.1) | 235 (23.2) |
| Summer | 1108 (27.7) | 168 (25.5) | 338 (26.2) | 304 (29.1) | 298 (29.5) |
| Autumn | 1152 (28.8) | 199 (30.2) | 376 (29.2) | 300 (28.7) | 277 (27.4) |
| Winter | 895 (22.4) | 151 (22.9) | 322 (25.0) | 221 (21.1) | 201 (19.9) |
| **Wear duration, day, mean (SD)** | 6.7 (0.7) | 6.7 (0.7) | 6.7 (0.6) | 6.7 (0.7) | 6.7 (0.7) |
| **Diabetes duration, year, mean (SD)** | 11.2 (9.4) | 11.5 (8.6) | 11.3 (9.1) | 10.9 (9.4) | 11.4 (10.3) |
| **HbA1c, mean (SD)** | 50.9 (13.0) | 51.5 (13.2) | 51.4 (13.6) | 50.3 (12.5) | 50.5 (12.7) |
| **Insulin medication use, n (%)** | 601 (15.1) | 94 (14.3) | 185 (14.4) | 145 (13.9) | 177 (17.6) |
| **Self-rated health, n (%)** |  |  |  |  |  |
| Excellent | 200 (5.0) | 22 (3.3) | 56 (4.3) | 48 (4.6) | 74 (7.3) |
| Good | 1851 (46.2) | 216 (32.8) | 558 (43.3) | 524 (50.1) | 553 (54.7) |
| Fair | 1476 (36.9) | 285 (43.2) | 492 (38.2) | 379 (36.3) | 320 (31.7) |
| poor | 476 (11.9) | 136 (20.6) | 182 (14.1) | 94 (9.0) | 64 (6.3) |
| **History of cancer or CVD, n (%)** | 1201 (30.0) | 278 (42.2) | 435 (33.8) | 291 (27.8) | 197 (19.5) |
| **History of hypertension, n (%)** | 2308 (57.7) | 452 (68.6) | 834 (64.8) | 549 (52.5) | 473 (46.8) |
| **Long-standing illness, disability or infirmity, n (%)** | 2980 (74.4) | 552 (83.8) | 982 (76.2) | 750 (71.8) | 696 (68.8) |
| **Illness, injury, bereavement, stress in last 2 years, n (%)** | 1962 (49.0) | 350 (53.1) | 640 (49.7) | 493 (47.2) | 479 (47.4) |

BMI indicates body mass index; CVD, cardiovascular disease; LPA, light-intensity physical activity; MPA, moderate-intensity physical activity; VPA, vigorous-intensity physical activity; MVPA, moderate-to-vigorous-intensity physical activity; SD, standard deviation.

**Supplementary Table 4. Baseline characteristics of 4003 participants by VPA in the UK Biobank.**

| **Characteristics** | **Total** | **Device-measured VPA, minutes/week** | | | |
| --- | --- | --- | --- | --- | --- |
|  |  | **0** | **1-14** | **15-29** | **≥30** |
| **Total, n** | 4003 | 1348 | 1316 | 845 | 494 |
| **Age, year, mean (SD)** | 64.9 (6.9) | 67.0 (6.1) | 64.7 (6.8) | 63.6 (7.0) | 61.8 (7.4) |
| **Sex, male, n (%)** | 2523 (63.0) | 760 (56.4) | 819 (62.2) | 603 (71.4) | 341 (69.0) |
| **Ethnicity, white, n (%)** | 3740 (93.4) | 1290 (95.7) | 1227 (93.2) | 786 (93.0) | 437 (88.5) |
| **Education, college or university, n (%)** | 1332 (33.3) | 420 (31.2) | 445 (33.8) | 275 (32.5) | 192 (38.9) |
| **BMI, kg/m^2^, mean (SD)** | 31.1 (5.8) | 32.5 (6.3) | 31.1 (5.7) | 30.1 (5.0) | 28.5 (4.8) |
| **Waist circumference, cm, mean (SD)** | 102.0 (14.4) | 105.4 (14.7) | 102.1 (14.1) | 100.0 (13.5) | 95.5 (13.2) |
| **Smoking status, n (%)** |  |  |  |  |  |
| Never | 1760 (44.0) | 558 (41.4) | 591 (44.9) | 365 (43.2) | 246 (49.8) |
| Former | 1905 (47.6) | 657 (48.7) | 615 (46.7) | 412 (48.8) | 221 (44.7) |
| Current | 338 (8.4) | 133 (9.9) | 110 (8.4) | 68 (8.0) | 27 (5.5) |
| **Diet score, mean (SD)** | 3.7 (1.9) | 3.7 (1.9) | 3.7 (1.8) | 3.8 (2.0) | 3.9 (1.8) |
| **Sleep score, mean (SD)** | 2.8 (1.0) | 2.7 (1.0) | 2.9 (1.0) | 2.9 (1.1) | 3.1 (1.0) |
| **Alcohol intake, g/day, mean (SD)** | 16.8 (21.6) | 14.3 (20.5) | 16.2 (20.8) | 20.3 (24.1) | 19.6 (21.1) |
| **LPA, minutes/week, mean (SD)** | 1901.3 (478.0) | 1701.1 (488.9) | 1936.0 (450.9) | 2057.2 (410.7) | 2088.8 (425.2) |
| **MPA, minutes/week, mean (SD)** | 340.8 (206.5) | 190.9 (120.0) | 332.6 (147.8) | 452.0 (177.5) | 581.4 (232.0) |
| **MVPA, minutes/week, mean (SD)** | 357.5 (220.7) | 190.9 (120.0) | 342.7 (147.8) | 475.3 (178.3) | 650.0 (239.1) |
| **Season of wear, n (%)** |  |  |  |  |  |
| Spring | 848 (21.2) | 281 (20.8) | 273 (20.7) | 186 (22.0) | 108 (21.9) |
| Summer | 1108 (27.7) | 362 (26.9) | 359 (27.3) | 250 (29.6) | 137 (27.7) |
| Autumn | 1152 (28.8) | 398 (29.5) | 387 (29.4) | 225 (26.6) | 142 (28.7) |
| Winter | 895 (22.4) | 307 (22.8) | 297 (22.6) | 184 (21.8) | 107 (21.7) |
| **Wear duration, day, mean (SD)** | 6.7 (0.7) | 6.7 (0.7) | 6.7 (0.6) | 6.7 (0.7) | 6.6 (0.7) |
| **Diabetes duration, year, mean (SD)** | 11.2 (9.4) | 11.9 (9.1) | 10.8 (9.5) | 10.6 (9.1) | 11.6 (10.6) |
| **HbA1c, mean (SD)** | 50.9 (13.0) | 51.6 (12.8) | 50.8 (13.2) | 50.5 (13.6) | 50.0 (12.0) |
| **Insulin medication use, n (%)** | 601 (15.1) | 201 (14.9) | 176 (13.4) | 132 (15.7) | 92 (18.7) |
| **Self-rated health, n (%)** |  |  |  |  |  |
| Excellent | 200 (5.0) | 48 (3.6) | 45 (3.4) | 65 (7.7) | 42 (8.5) |
| Good | 1851 (46.2) | 533 (39.5) | 622 (47.3) | 417 (49.3) | 279 (56.5) |
| Fair | 1476 (36.9) | 549 (40.7) | 487 (37.0) | 290 (34.3) | 150 (30.4) |
| poor | 476 (11.9) | 218 (16.2) | 162 (12.3) | 73 (8.6) | 23 (4.7) |
| **History of cancer or CVD, n (%)** | 1201 (30.0) | 516 (38.3) | 366 (27.8) | 223 (26.4) | 96 (19.4) |
| **History of hypertension, n (%)** | 2308 (57.7) | 887 (65.8) | 776 (59.0) | 422 (49.9) | 223 (45.1) |
| **Long-standing illness, disability or infirmity, n (%)** | 2980 (74.4) | 1083 (80.3) | 977 (74.2) | 585 (69.2) | 335 (67.8) |
| **Illness, injury, bereavement, stress in last 2 years, n (%)** | 1962 (49.0) | 677 (50.2) | 677 (51.4) | 391 (46.3) | 217 (43.9) |

BMI indicates body mass index; CVD, cardiovascular disease; LPA, light-intensity physical activity; MPA, moderate-intensity physical activity; VPA, vigorous-intensity physical activity; MVPA, moderate-to-vigorous-intensity physical activity; SD, standard deviation.

**Supplementary Table 5. Population-attributable fractions (PAFs) for all-cause mortality associated with physical activity.**

| **Physical activity categories** | **Proportions of the study sample, %** | **PAF** |
| --- | --- | --- |
|  |  | **Percent (95% CI)** |
| **LPA (minutes/week)** |  |  |
| < 1750 | 37.8 | 18.75 (2.46-32.32) |
| 1750-2099 | 29.3 | 9.13 (0.00-18.52) |
| 2100-2450 | 20.5 | 0.00 (0.00-5.87) |
| **MPA (minutes/week)** |  |  |
| < 150 | 16.5 | 28.04 (20.79-34.63) |
| 150-300 | 32.2 | 22.39 (13.79-30.14) |
| 300-450 | 26.1 | 6.95 (1.13-12.43) |
| **VPA (minutes/week)** |  |  |
| 0 | 33.7 | 31.06 (15.56-43.71) |
| 1-14 | 32.9 | 6.13 (0.00-15.74) |
| 15-30 | 21.1 | 2.24 (0.00-8.37) |
| **MVPA (minutes/week)** |  |  |
| < 275 | 41.6 | 47.09 (30.07-59.96) |
| 275-449 | 29.9 | 8.79 (0.10-16.72) |
| 450-625 | 17.1 | 1.01 (0.00-5.32) |

We utilized diabetic participants (n = 4,003) from the UK Biobank with valid accelerometer data in the analyses.

Population-attributable fractions below 0 were truncated at 0.

LPA, light-intensity physical activity; MPA, moderate-intensity physical activity; VPA, vigorous-intensity physical activity; MVPA, moderate-to-vigorous-intensity physical activity. The widths of the lines extending from the center points represent 95% CI.

**Supplementary Table 6. Hazard ratios (95% CI) for cancer mortality according to physical activity among individuals with type 2 diabetes.**

| **Exposures** | **Cancer mortality** | **Incidence rate per 1000 person-year** | **HR (95% CI) *** | | |
| --- | --- | --- | --- | --- | --- |
|  | **No. of cases** |  | **Model 1** | **Model 2** | **Model 3** |
| **LPA (minutes/week)** |  |  |  |  |  |
| <1750 | 70 | 6.98 | 1.00 (ref) | 1.00 (ref) | 1.00 (ref) |
| 1750-2099 | 54 | 6.84 | 1.03 (0.72,1.47) | 1.05 (0.73,1.50) | 1.09 (0.76,1.57) |
| 2100-2449 | 19 | 3.39 | 0.53 (0.32,0.88) | 0.55 (0.33,0.92) | 0.57 (0.34,0.96) |
| ≥2450 | 15 | 4.49 | 0.76 (0.43,1.33) | 0.76 (0.43,1.34) | 0.80 (0.45,1.42) |
| ***p-value* for trend** | | | =0.055 | =0.072 | =0.121 |
| **MPA (minutes/week)** |  |  |  |  |  |
| <150 | 43 | 10.28 | 1.00 (ref) | 1.00 (ref) | 1.00 (ref) |
| 150-299 | 57 | 6.64 | 0.71 (0.48,1.06) | 0.73 (0.49,1.10) | 0.73 (0.49,1.10) |
| 300-449 | 40 | 5.64 | 0.65 (0.42,1.00) | 0.69 (0.44,1.07) | 0.70 (0.44,1.11) |
| ≥450 | 18 | 2.57 | 0.34 (0.19,0.59) | 0.36 (0.20,0.63) | 0.37 (0.20,0.67) |
| ***p-value* for trend** | | | <0.001 | =0.001 | =0.002 |
| **VPA (minutes/week)** |  |  |  |  |  |
| 0 | 76 | 8.68 | 1.00 (ref) | 1.00 (ref) | 1.00 (ref) |
| 1-14 | 38 | 4.26 | 0.54 (0.37,0.81) | 0.55 (0.37,0.82) | 0.58 (0.39,0.86) |
| 15-29 | 32 | 5.53 | 0.75 (0.49,1.14) | 0.76 (0.50,1.17) | 0.81 (0.52,1.25) |
| ≥30 | 12 | 3.52 | 0.54 (0.29,1.00) | 0.57 (0.30,1.06) | 0.61 (0.32,1.17) |
| ***p-value* for trend** | | | =0.053 | =0.084 | =0.158 |
| **MVPA (minutes/week)** |  |  |  |  |  |
| <275 | 93 | 8.57 | 1.00 (ref) | 1.00 (ref) | 1.00 (ref) |
| 275-449 | 42 | 5.18 | 0.67 (0.46,0.97) | 0.70 (0.48,1.01) | 0.72 (0.50,1.06) |
| 450-624 | 18 | 3.80 | 0.54 (0.32,0.90) | 0.55 (0.33,0.92) | 0.57 (0.34,0.96) |
| ≥625 | 5 | 1.57 | 0.25 (0.10,0.61) | 0.26 (0.11,0.66) | 0.27 (0.11,0.69) |
| ***p-value* for trend** | | | <0.001 | <0.001 | =0.001 |

* Hazard ratios (95% CI) were calculated in Cox proportional hazards model: model 1, adjusted for age (years), sex (male or female), ethnicity (white or others), education (college/university or others), season at the time of accelerometry recording (spring, summer, autumn, or winter), and accelerometer wear duration (days); model 2, further adjusted for smoking status (never, former, or current), alcohol intake (g/day), diet score (0 to 7), and sleep score (0 to 5) based on model 1; model 3, further adjusted for body mass index (kg/m^2^), waist circumference (cm), self-rated health (excellent, good, fair, or poor), long-standing illness, disability or infirmity (yes or no), illness, injury, bereavement, or stress in last 2 years (yes or no), history of cancer or cardiovascular disease (yes or no), history of hypertension (yes or no), and diabetes duration (years) based on model 2. Wald tests were used to obtain the two-sided *p-value*.

HR indicates hazard ratio; CI, confidence interval; LPA, light-intensity physical activity; MPA, moderate-intensity physical activity; VPA, vigorous-intensity physical activity; MVPA, moderate-to-vigorous-intensity physical activity.

**Supplementary Table 7. Hazard ratios (95% CI) for cardiovascular-disease mortality according to physical activity among individuals with type 2 diabetes.**

| **Exposures** | **Cardiovascular**  **disease mortality** | **Incidence rate per 1000 person-year** | **HR (95% CI) *** | | |
| --- | --- | --- | --- | --- | --- |
|  | **No. of cases** |  | **Model 1** | **Model 2** | **Model 3** |
| **LPA (minutes/week)** |  |  |  |  |  |
| <1750 | 47 | 4.69 | 1.00 (ref) | 1.00 (ref) | 1.00 (ref) |
| 1750-2099 | 19 | 2.41 | 0.55 (0.32,0.94) | 0.54 (0.31,0.92) | 0.64 (0.37,1.10) |
| 2100-2449 | 14 | 2.50 | 0.62 (0.34,1.13) | 0.61 (0.33,1.12) | 0.74 (0.40,1.36) |
| ≥2450 | 3 | 0.90 | 0.25 (0.08,0.81) | 0.25 (0.08,0.80) | 0.31 (0.09,1.02) |
| ***p-value* for trend** | | | =0.004 | =0.003 | =0.032 |
| **MPA (minutes/week)** |  |  |  |  |  |
| <150 | 28 | 6.70 | 1.00 (ref) | 1.00 (ref) | 1.00 (ref) |
| 150-299 | 37 | 4.31 | 0.71 (0.43,1.17) | 0.69 (0.42,1.13) | 0.81 (0.48,1.35) |
| 300-449 | 11 | 1.55 | 0.29 (0.14,0.58) | 0.28 (0.14,0.56) | 0.37 (0.18,0.77) |
| ≥450 | 7 | 1.00 | 0.21 (0.09,0.50) | 0.21 (0.09,0.49) | 0.32 (0.13,0.79) |
| ***p-value* for trend** | | | <0.001 | <0.001 | =0.002 |
| **VPA (minutes/week)** |  |  |  |  |  |
| 0 | 42 | 4.80 | 1.00 (ref) | 1.00 (ref) | 1.00 (ref) |
| 1-14 | 31 | 3.47 | 0.80 (0.50,1.27) | 0.80 (0.50,1.28) | 0.99 (0.61,1.60) |
| 15-29 | 7 | 1.21 | 0.28 (0.13,0.63) | 0.28 (0.12,0.62) | 0.38 (0.17,0.88) |
| ≥30 | 3 | 0.88 | 0.24 (0.07,0.78) | 0.23 (0.07,0.76) | 0.35 (0.10,1.15) |
| ***p-value* for trend** | | | =0.001 | =0.001 | =0.021 |
| **MVPA (minutes/week)** |  |  |  |  |  |
| <275 | 59 | 5.43 | 1.00 (ref) | 1.00 (ref) | 1.00 (ref) |
| 275-449 | 16 | 1.97 | 0.42 (0.24,0.73) | 0.41 (0.23,0.72) | 0.50 (0.28,0.89) |
| 450-624 | 5 | 1.06 | 0.25 (0.10,0.61) | 0.24 (0.09,0.60) | 0.32 (0.13,0.82) |
| ≥625 | 3 | 0.94 | 0.26 (0.08,0.83) | 0.26 (0.08,0.83) | 0.38 (0.11,1.29) |
| ***p-value* for trend** | | | <0.001 | <0.001 | =0.004 |

***** Hazard ratios (95% CI) were calculated in Cox proportional hazards model: model 1, adjusted for age (years), sex (male or female), ethnicity (white or others), education (college/university or others), season at the time of accelerometry recording (spring, summer, autumn, or winter), and accelerometer wear duration (days); model 2, further adjusted for smoking status (never, former, or current), alcohol intake (g/day), diet score (0 to 7), and sleep score (0 to 5) based on model 1; model 3, further adjusted for body mass index (kg/m^2^), waist circumference (cm), self-rated health (excellent, good, fair, or poor), long-standing illness, disability or infirmity (yes or no), illness, injury, bereavement, or stress in last 2 years (yes or no), history of cancer or cardiovascular disease (yes or no), history of hypertension (yes or no), and diabetes duration (years) based on model 2. Wald tests were used to obtain the two-sided *p-value*.

HR indicates hazard ratio; CI, confidence interval; LPA, light-intensity physical activity; MPA, moderate-intensity physical activity; VPA, vigorous-intensity physical activity; MVPA, moderate-to-vigorous-intensity physical activity.

**Supplementary Table 8. Joint association of LPA and MPA with all-cause and cause-specific mortality for the risk matrix.**

| **Exposure** | | **No. of individuals** | **HR (95% CI)** | | |
| --- | --- | --- | --- | --- | --- |
| **LPA (minutes/week)** | **MPA (minutes/week)** | **Total = 4,003** | **All-cause**  **mortality** | **Cancer**  **mortality** | **Cardiovascular**  **disease mortality** |
| < 1750 | < 150 | 526 | 1.00 (ref) | 1.00 (ref) | 1.00 (ref) |
| < 1750 | 150-299 | 585 | 0.54 (0.38,0.76) | 0.72 (0.43,1.21) | 0.74 (0.40,1.39) |
| < 1750 | 300-449 | 274 | 0.27 (0.14,0.51) | 0.51 (0.23,1.12) | 0.32 (0.09,1.09) |
| < 1750 | ≥ 450 | 130 | 0.11 (0.03,0.44) | 0.13 (0.02,0.99) | 0.25 (0.03,1.90) |
| 1750-2099 | < 150 | 100 | 0.77 (0.44,1.33) | 0.87 (0.36,2.10) | 0.59 (0.18,1.99) |
| 1750-2099 | 150-299 | 416 | 0.65 (0.45,0.93) | 0.85 (0.49,1.47) | 0.53 (0.24,1.16) |
| 1750-2099 | 300-449 | 381 | 0.46 (0.30,0.72) | 0.80 (0.44,1.46) | 0.33 (0.11,0.97) |
| 1750-2099 | ≥ 450 | 276 | 0.39 (0.22,0.70) | 0.57 (0.25,1.30) | 0.41 (0.12,1.45) |
| 2100-2449 | < 150 | 26 | 0.65 (0.24,1.79) | 0.86 (0.20,3.65) | - * |
| 2100-2449 | 150-299 | 203 | 0.56 (0.34,0.91) | 0.38 (0.15,0.97) | 1.13 (0.52,2.47) |
| 2100-2449 | 300-449 | 255 | 0.35 (0.19,0.63) | 0.57 (0.26,1.27) | 0.23 (0.05,1.00) |
| 2100-2449 | ≥ 450 | 338 | 0.19 (0.09,0.39) | 0.24 (0.08,0.68) | 0.36 (0.10,1.25) |
| ≥ 2450 | < 150 | 7 | - * | - * | - * |
| ≥ 2450 | 150-299 | 84 | 0.59 (0.30,1.19) | 0.73 (0.25,2.10) | 0.29 (0.04,2.16) |
| ≥ 2450 | 300-449 | 135 | 0.51 (0.26,1.00) | 0.80 (0.33,1.93) | 0.46 (0.11,1.99) |
| ≥ 2450 | ≥ 450 | 267 | 0.19 (0.08,0.44) | 0.38 (0.15,1.02) | - * |

* There were not enough participants in these categories for analysis.

Hazard ratios were calculated in Cox proportional hazards model after adjusting for age (years), sex (male or female), ethnicity (white or others), education (college/university or others), season at the time of accelerometry recording (spring, summer, autumn, or winter), accelerometer wear duration (days), smoking status (never, former, or current), alcohol intake (g/day), diet score (0 to 7), sleep score (0 to 5), body mass index (kg/m^2^), waist circumference (cm), self-rated health (excellent, good, fair, or poor), long-standing illness, disability or infirmity (yes or no), illness, injury, bereavement, or stress in last 2 years (yes or no), history of cancer or cardiovascular disease (yes or no), history of hypertension (yes or no), and diabetes duration (years).

HR indicates hazard ratio; CI, confidence interval; LPA, light-intensity physical activity; MPA, moderate-intensity physical activity; VPA, vigorous-intensity physical activity; MVPA, moderate-to-vigorous-intensity physical activity.

**Supplementary Table 9. Joint association of LPA and VPA with all-cause and cause-specific mortality for the risk matrix.**

| **Exposure** | | **No. of individuals** | **HR (95% CI)** | | |
| --- | --- | --- | --- | --- | --- |
| **LPA (minutes/week)** | **VPA (minutes/week)** | **Total = 4003** | **All-cause mortality** | **Cancer**  **mortality** | **Cardiovascular**  **disease mortality** |
| < 1750 | 0 | 756 | 1.00 (ref) | 1.00 (ref) | 1.00 (ref) |
| < 1750 | 1-14 | 462 | 0.48 (0.32,0.71) | 0.48 (0.26,0.89) | 0.71 (0.35,1.41) |
| < 1750 | 15-29 | 195 | 0.43 (0.23,0.80) | 0.63 (0.28,1.44) | 0.75 (0.26,2.22) |
| < 1750 | ≥ 30 | 102 | 0.17 (0.04,0.68) | 0.37 (0.09,1.57) | - * |
| 1750-2099 | 0 | 334 | 0.69 (0.47,1.00) | 0.83 (0.48,1.44) | 0.30 (0.11,0.87) |
| 1750-2099 | 1-14 | 411 | 0.70 (0.48,1.01) | 0.79 (0.45,1.38) | 1.12 (0.57,2.21) |
| 1750-2099 | 15-29 | 269 | 0.64 (0.40,1.03) | 1.17 (0.64,2.12) | 0.12 (0.02,0.91) |
| 1750-2099 | ≥ 30 | 159 | 0.43 (0.20,0.95) | 0.27 (0.06,1.15) | 0.26 (0.03,1.98) |
| 2100-2449 | 0 | 177 | 0.68 (0.41,1.12) | 0.56 (0.24,1.32) | 0.93 (0.38,2.27) |
| 2100-2449 | 1-14 | 268 | 0.33 (0.17,0.63) | 0.30 (0.11,0.84) | 0.59 (0.21,1.71) |
| 2100-2449 | 15-29 | 241 | 0.40 (0.22,0.72) | 0.45 (0.19,1.08) | 0.31 (0.07,1.32) |
| 2100-2449 | ≥ 30 | 136 | 0.35 (0.14,0.88) | 0.48 (0.14,1.58) | 0.58 (0.13,2.54) |
| ≥ 2450 | 0 | 81 | 0.63 (0.29,1.37) | 0.87 (0.31,2.45) | 0.40 (0.05,3.00) |
| ≥ 2450 | 1-14 | 175 | 0.40 (0.20,0.79) | 0.30 (0.09,0.96) | 0.42 (0.10,1.80) |
| ≥ 2450 | 15-29 | 140 | 0.27 (0.10,0.73) | 0.44 (0.13,1.45) | - * |
| ≥ 2450 | ≥ 30 | 97 | 0.67 (0.27,1.69) | 1.55 (0.58,4.12) | - * |

* There were not enough participants in these categories for analysis.

Hazard ratios were calculated in Cox proportional hazards model after adjusting for age (years), sex (male or female), ethnicity (white or others), education (college/university or others), season at the time of accelerometry recording (spring, summer, autumn, or winter), accelerometer wear duration (days), smoking status (never, former, or current), alcohol intake (g/day), diet score (0 to 7), sleep score (0 to 5), body mass index (kg/m^2^), waist circumference (cm), self-rated health (excellent, good, fair, or poor), long-standing illness, disability or infirmity (yes or no), illness, injury, bereavement, or stress in last 2 years (yes or no), history of cancer or cardiovascular disease (yes or no), history of hypertension (yes or no), and diabetes duration (years).

HR indicates hazard ratio; CI, confidence interval; LPA, light-intensity physical activity; MPA, moderate-intensity physical activity; VPA, vigorous-intensity physical activity; MVPA, moderate-to-vigorous-intensity physical activity.

**Supplementary Table 10. Joint association of MPA and VPA with all-cause and cause-specific mortality for the risk matrix.**

| **Exposure** | | **No. of individuals** | **HR (95% CI)** | | |
| --- | --- | --- | --- | --- | --- |
| **MPA (minutes/week)** | **VPA (minutes/week)** | **Total = 4003** | **All-cause mortality** | **Cancer**  **mortality** | **Cardiovascular disease mortality** |
| < 150 | 0 | 567 | 1.00 (ref) | 1.00 (ref) | 1.00 (ref) |
| < 150 | 1-14 | 82 | 0.64 (0.34,1.21) | 0.15 (0.02,1.12) | 1.46 (0.55,3.91) |
| < 150 | 15-29 | 9 | - * | - * | - * |
| < 150 | ≥ 30 | 1 | - * | - * | - * |
| 150-299 | 0 | 579 | 0.65 (0.48,0.89) | 0.71 (0.44,1.16) | 0.85 (0.45,1.61) |
| 150-299 | 1-14 | 519 | 0.56 (0.39,0.79) | 0.60 (0.35,1.02) | 0.95 (0.49,1.85) |
| 150-299 | 15-29 | 154 | 0.43 (0.24,0.80) | 0.53 (0.22,1.27) | 0.70 (0.23,2.08) |
| 150-299 | ≥ 30 | 36 | 0.37 (0.09,1.54) | 0.45 (0.06,3.35) | - * |
| 300-449 | 0 | 151 | 0.34 (0.17,0.71) | 0.50 (0.19,1.27) | 0.44 (0.10,1.91) |
| 300-449 | 1-14 | 488 | 0.35 (0.22,0.54) | 0.46 (0.24,0.86) | 0.55 (0.23,1.33) |
| 300-449 | 15-29 | 289 | 0.47 (0.29,0.77) | 0.79 (0.42,1.50) | 0.27 (0.06,1.17) |
| 300-449 | ≥ 30 | 117 | 0.44 (0.21,0.91) | 0.89 (0.39,2.05) | - * |
| ≥ 450 | 0 | 51 | - * | - * | - * |
| ≥ 450 | 1-14 | 227 | 0.16 (0.07,0.41) | 0.15 (0.03,0.62) | 0.62 (0.18,2.18) |
| ≥ 450 | 15-29 | 393 | 0.30 (0.17,0.52) | 0.52 (0.26,1.03) | 0.11 (0.01,0.86) |
| ≥ 450 | ≥ 30 | 340 | 0.23 (0.11,0.48) | 0.23 (0.08,0.69) | 0.50 (0.14,1.81) |

* There were not enough participants in these categories for analysis.

Hazard ratios were calculated in Cox proportional hazards model after adjusting for age (years), sex (male or female), ethnicity (white or others), education (college/university or others), season at the time of accelerometry recording (spring, summer, autumn, or winter), accelerometer wear duration (days), smoking status (never, former, or current), alcohol intake (g/day), diet score (0 to 7), sleep score (0 to 5), body mass index (kg/m^2^), waist circumference (cm), self-rated health (excellent, good, fair, or poor), long-standing illness, disability or infirmity (yes or no), illness, injury, bereavement, or stress in last 2 years (yes or no), history of cancer or cardiovascular disease (yes or no), history of hypertension (yes or no), and diabetes duration (years).

HR indicates hazard ratio; CI, confidence interval; LPA, light-intensity physical activity; MPA, moderate-intensity physical activity; VPA, vigorous-intensity physical activity; MVPA, moderate-to-vigorous-intensity physical activity.

**Supplementary Table 11. Association between LPA and all-cause mortality among participants with type 2 diabetes in subgroups** *.

| **Subgroups** | **LPA, minutes/week** | | | | ***p-value* for interaction** |
| --- | --- | --- | --- | --- | --- |
|  | **<1750** | **1750-2099** | **2100-2449** | **≥2450** |  |
| **Age** |  |  |  |  | **0.4682** |
| < 60 | 1.00 (ref) | 1.81 (0.64,5.13) | 1.51 (0.50,4.61) | 0.39 (0.05,3.36) |  |
| ≥ 60 | 1.00 (ref) | 0.85 (0.65,1.10) | 0.55 (0.38,0.79) | 0.62 (0.40,0.97) |  |
| **Sex** |  |  |  |  | **0.5894** |
| Female | 1.00 (ref) | 0.79 (0.49,1.28) | 0.63 (0.36,1.12) | 0.48 (0.23,1.01) |  |
| Male | 1.00 (ref) | 0.96 (0.71,1.30) | 0.59 (0.38,0.90) | 0.73 (0.43,1.25) |  |
| **BMI** |  |  |  |  | **0.9953** |
| < 25 | 1.00 (ref) | 0.86 (0.33,2.26) | 0.30 (0.08,1.13) | 0.60 (0.17,2.17) |  |
| ≥ 25 | 1.00 (ref) | 0.90 (0.69,1.17) | 0.62 (0.43,0.88) | 0.62 (0.39,0.99) |  |
| **Waist** |  |  |  |  | **0.3053** |
| Healthy | 1.00 (ref) | 1.12 (0.72,1.74) | 0.57 (0.31,1.03) | 0.60 (0.29,1.21) |  |
| Unhealthy | 1.00 (ref) | 0.79 (0.58,1.09) | 0.66 (0.44,1.00) | 0.68 (0.39,1.18) |  |
| **Smoking status** |  |  |  |  | **0.3449** |
| Never | 1.00 (ref) | 0.85 (0.52,1.38) | 0.71 (0.41,1.22) | 0.34 (0.13,0.86) |  |
| Ever | 1.00 (ref) | 0.93 (0.69,1.26) | 0.56 (0.36,0.87) | 0.80 (0.49,1.32) |  |
| **Alcohol intake** |  |  |  |  | **0.6354** |
| Healthy | 1.00 (ref) | 0.94 (0.70,1.25) | 0.61 (0.41,0.91) | 0.52 (0.31,0.89) |  |
| Unhealthy | 1.00 (ref) | 0.82 (0.48,1.39) | 0.62 (0.32,1.23) | 0.99 (0.46,2.16) |  |
| **Diet score** |  |  |  |  | **0.9719** |
| < 4 | 1.00 (ref) | 0.91 (0.64,1.28) | 0.62 (0.39,0.99) | 0.69 (0.37,1.28) |  |
| ≥ 4 | 1.00 (ref) | 0.96 (0.66,1.39) | 0.63 (0.39,1.03) | 0.59 (0.32,1.08) |  |
| **Sleep score** |  |  |  |  | **0.9636** |
| < 3 | 1.00 (ref) | 0.93 (0.60,1.44) | 0.67 (0.38,1.18) | 0.61 (0.26,1.46) |  |
| ≥ 3 | 1.00 (ref) | 0.90 (0.66,1.23) | 0.61 (0.40,0.93) | 0.67 (0.41,1.12) |  |
| **History of hypertension** |  |  |  |  | **0.3645** |
| No | 1.00 (ref) | 0.71 (0.46,1.09) | 0.50 (0.28,0.89) | 0.59 (0.31,1.13) |  |
| Yes | 1.00 (ref) | 1.04 (0.76,1.43) | 0.74 (0.49,1.12) | 0.63 (0.35,1.13) |  |

* Hazard ratios were calculated in Cox proportional hazards model after adjusting for age (years), sex (male or female), ethnicity (white or others), education (college/university or others), season at the time of accelerometry recording (spring, summer, autumn, or winter), accelerometer wear duration (days), smoking status (never, former, or current), alcohol intake (g/day), diet score (0 to 7), sleep score (0 to 5), body mass index (kg/m^2^), waist circumference (cm), self-rated health (excellent, good, fair, or poor), long-standing illness, disability or infirmity (yes or no), illness, injury, bereavement, or stress in last 2 years (yes or no), history of cancer or cardiovascular disease (yes or no), history of hypertension (yes or no), and diabetes duration (years). Wald tests were used to obtain the two-sided *p-value*.

HR indicates hazard ratio; CI, confidence interval; LPA, light-intensity physical activity; MPA, moderate-intensity physical activity; VPA, vigorous-intensity physical activity; MVPA, moderate-to-vigorous-intensity physical activity.

**Supplementary Table 12. Association between MPA and all-cause mortality among participants with type 2 diabetes in subgroups** *.

| **Subgroups** | **MPA, minutes/week** | | | | ***p-value* for interaction** |
| --- | --- | --- | --- | --- | --- |
|  | **<150** | **150-299** | **300-449** | **≥450** |  |
| **Age** |  |  |  |  | **0.4678** |
| < 60 | 1.00 (ref) | 0.37 (0.12,1.15) | 0.31 (0.09,1.08) | 0.11 (0.02,0.54) |  |
| ≥ 60 | 1.00 (ref) | 0.59 (0.45,0.77) | 0.38 (0.27,0.53) | 0.23 (0.15,0.37) |  |
| **Sex** |  |  |  |  | **0.5860** |
| Female | 1.00 (ref) | 0.74 (0.46,1.20) | 0.33 (0.17,0.63) | 0.29 (0.13,0.63) |  |
| Male | 1.00 (ref) | 0.59 (0.43,0.81) | 0.46 (0.32,0.68) | 0.24 (0.14,0.41) |  |
| **BMI** |  |  |  |  | **0.7982** |
| < 25 | 1.00 (ref) | 0.19 (0.06,0.53) | 0.10 (0.03,0.30) | 0.07 (0.02,0.27) |  |
| ≥ 25 | 1.00 (ref) | 0.64 (0.49,0.84) | 0.44 (0.31,0.62) | 0.27 (0.17,0.43) |  |
| **Waist** |  |  |  |  | **0.2523** |
| Healthy | 1.00 (ref) | 0.39 (0.23,0.64) | 0.30 (0.17,0.54) | 0.22 (0.11,0.42) |  |
| Unhealthy | 1.00 (ref) | 0.72 (0.53,0.98) | 0.46 (0.30,0.69) | 0.22 (0.11,0.41) |  |
| **Smoking status** |  |  |  |  | **0.9942** |
| Never | 1.00 (ref) | 0.62 (0.38,1.03) | 0.40 (0.22,0.73) | 0.22 (0.10,0.47) |  |
| Ever | 1.00 (ref) | 0.62 (0.46,0.84) | 0.41 (0.28,0.62) | 0.26 (0.15,0.43) |  |
| **Alcohol intake** |  |  |  |  | **0.4036** |
| Healthy | 1.00 (ref) | 0.62 (0.46,0.83) | 0.36 (0.24,0.53) | 0.21 (0.13,0.37) |  |
| Unhealthy | 1.00 (ref) | 0.62 (0.34,1.15) | 0.59 (0.30,1.18) | 0.35 (0.15,0.79) |  |
| **Diet score** |  |  |  |  | **0.2262** |
| < 4 | 1.00 (ref) | 0.56 (0.39,0.81) | 0.50 (0.32,0.77) | 0.25 (0.14,0.46) |  |
| ≥ 4 | 1.00 (ref) | 0.73 (0.50,1.07) | 0.35 (0.21,0.58) | 0.25 (0.14,0.47) |  |
| **Sleep score** |  |  |  |  | **0.2328** |
| < 3 | 1.00 (ref) | 0.67 (0.43,1.05) | 0.50 (0.29,0.88) | 0.47 (0.24,0.92) |  |
| ≥ 3 | 1.00 (ref) | 0.60 (0.43,0.83) | 0.38 (0.25,0.57) | 0.18 (0.10,0.31) |  |
| **History of hypertension** |  |  |  |  | **0.0977** |
| No | 1.00 (ref) | 0.47 (0.30,0.74) | 0.33 (0.19,0.56) | 0.14 (0.07,0.29) |  |
| Yes | 1.00 (ref) | 0.72 (0.52,1.00) | 0.49 (0.32,0.76) | 0.37 (0.22,0.64) |  |

* Hazard ratios were calculated in Cox proportional hazards model after adjusting for age (years), sex (male or female), ethnicity (white or others), education (college/university or others), season at the time of accelerometry recording (spring, summer, autumn, or winter), accelerometer wear duration (days), smoking status (never, former, or current), alcohol intake (g/day), diet score (0 to 7), sleep score (0 to 5), body mass index (kg/m^2^), waist circumference (cm), self-rated health (excellent, good, fair, or poor), long-standing illness, disability or infirmity (yes or no), illness, injury, bereavement, or stress in last 2 years (yes or no), history of cancer or cardiovascular disease (yes or no), history of hypertension (yes or no), and diabetes duration (years). Wald tests were used to obtain the two-sided *p-value*.

HR indicates hazard ratio; CI, confidence interval; LPA, light-intensity physical activity; MPA, moderate-intensity physical activity; VPA, vigorous-intensity physical activity; MVPA, moderate-to-vigorous-intensity physical activity.

**Supplementary Table 13. Association between VPA and all-cause mortality among participants with type 2 diabetes in subgroups** *.

| **Subgroups** | **VPA, minutes/week** | | | | ***p-value* for interaction** |
| --- | --- | --- | --- | --- | --- |
|  | **<0** | **1-14** | **15-29** | **≥30** |  |
| **Age** |  |  |  |  | **0.3087** |
| < 60 | 1.00 (ref) | 0.32 (0.09,1.09) | 0.78 (0.27,2.24) | 0.26 (0.05,1.41) |  |
| ≥ 60 | 1.00 (ref) | 0.57 (0.44,0.74) | 0.46 (0.32,0.66) | 0.42 (0.25,0.71) |  |
| **Sex** |  |  |  |  | **0.7332** |
| Female | 1.00 (ref) | 0.63 (0.39,1.03) | 0.70 (0.35,1.38) | 0.78 (0.31,1.91) |  |
| Male | 1.00 (ref) | 0.58 (0.42,0.79) | 0.49 (0.33,0.72) | 0.36 (0.20,0.66) |  |
| **BMI** |  |  |  |  | **0.4262** |
| < 25 | 1.00 (ref) | 0.63 (0.26,1.50) | 0.11 (0.02,0.65) | 0.43 (0.12,1.52) |  |
| ≥ 25 | 1.00 (ref) | 0.58 (0.44,0.77) | 0.59 (0.42,0.83) | 0.44 (0.26,0.77) |  |
| **Waist** |  |  |  |  | **0.1440** |
| Healthy | 1.00 (ref) | 0.84 (0.52,1.34) | 0.78 (0.46,1.31) | 0.46 (0.21,1.01) |  |
| Unhealthy | 1.00 (ref) | 0.51 (0.37,0.71) | 0.41 (0.26,0.67) | 0.49 (0.25,0.96) |  |
| **Smoking status** |  |  |  |  | **0.8331** |
| Never | 1.00 (ref) | 0.62 (0.38,1.00) | 0.46 (0.24,0.89) | 0.45 (0.19,1.06) |  |
| Ever | 1.00 (ref) | 0.58 (0.42,0.79) | 0.58 (0.39,0.86) | 0.44 (0.24,0.81) |  |
| **Alcohol intake** |  |  |  |  | **0.4142** |
| Healthy | 1.00 (ref) | 0.57 (0.42,0.77) | 0.43 (0.29,0.66) | 0.42 (0.23,0.78) |  |
| Unhealthy | 1.00 (ref) | 0.67 (0.38,1.18) | 0.83 (0.45,1.53) | 0.53 (0.22,1.27) |  |
| **Diet score** |  |  |  |  | **0.7935** |
| < 4 | 1.00 (ref) | 0.58 (0.40,0.83) | 0.55 (0.35,0.87) | 0.53 (0.27,1.01) |  |
| ≥ 4 | 1.00 (ref) | 0.61 (0.42,0.89) | 0.54 (0.33,0.89) | 0.38 (0.18,0.82) |  |
| **Sleep score** |  |  |  |  | **0.1853** |
| < 3 | 1.00 (ref) | 0.60 (0.37,0.97) | 0.81 (0.47,1.40) | 0.87 (0.39,1.92) |  |
| ≥ 3 | 1.00 (ref) | 0.58 (0.42,0.79) | 0.44 (0.28,0.67) | 0.32 (0.17,0.62) |  |
| **History of hypertension** |  |  |  |  | **0.5645** |
| No | 1.00 (ref) | 0.60 (0.39,0.93) | 0.47 (0.28,0.80) | 0.35 (0.15,0.80) |  |
| Yes | 1.00 (ref) | 0.61 (0.44,0.85) | 0.61 (0.40,0.94) | 0.57 (0.31,1.06) |  |

* Hazard ratios were calculated in Cox proportional hazards model after adjusting for age (years), sex (male or female), ethnicity (white or others), education (college/university or others), season at the time of accelerometry recording (spring, summer, autumn, or winter), accelerometer wear duration (days), smoking status (never, former, or current), alcohol intake (g/day), diet score (0 to 7), sleep score (0 to 5), body mass index (kg/m^2^), waist circumference (cm), self-rated health (excellent, good, fair, or poor), long-standing illness, disability or infirmity (yes or no), illness, injury, bereavement, or stress in last 2 years (yes or no), history of cancer or cardiovascular disease (yes or no), history of hypertension (yes or no), and diabetes duration (years). Wald tests were used to obtain the two-sided *p-value*.

HR indicates hazard ratio; CI, confidence interval; LPA, light-intensity physical activity; MPA, moderate-intensity physical activity; VPA, vigorous-intensity physical activity; MVPA, moderate-to-vigorous-intensity physical activity.

**Supplementary Table 14. Association between MVPA and all-cause mortality among participants with type 2 diabetes in subgroups** *.

| **Subgroups** | **MVPA, minutes/week** | | | | ***p-value* for interaction** |
| --- | --- | --- | --- | --- | --- |
|  | **<275** | **275-449** | **450-624** | **≥625** |  |
| **Age** |  |  |  |  | **0.9707** |
| < 60 | 1.00 (ref) | 0.68 (0.26,1.80) | 0.44 (0.11,1.79) | 0.32 (0.06,1.67) |  |
| ≥ 60 | 1.00 (ref) | 0.51 (0.38,0.67) | 0.34 (0.22,0.53) | 0.28 (0.15,0.54) |  |
| **Sex** |  |  |  |  | **0.3676** |
| Female | 1.00 (ref) | 0.42 (0.24,0.72) | 0.38 (0.18,0.81) | 0.56 (0.23,1.37) |  |
| Male | 1.00 (ref) | 0.60 (0.44,0.83) | 0.37 (0.22,0.61) | 0.22 (0.10,0.52) |  |
| **BMI** |  |  |  |  | **0.8915** |
| < 25 | 1.00 (ref) | 0.35 (0.13,0.91) | 0.23 (0.06,0.85) | 0.15 (0.03,0.77) |  |
| ≥ 25 | 1.00 (ref) | 0.56 (0.42,0.75) | 0.39 (0.25,0.61) | 0.36 (0.19,0.70) |  |
| **Waist** |  |  |  |  | **0.5840** |
| Healthy | 1.00 (ref) | 0.55 (0.34,0.87) | 0.45 (0.25,0.84) | 0.38 (0.18,0.82) |  |
| Unhealthy | 1.00 (ref) | 0.55 (0.39,0.78) | 0.33 (0.18,0.60) | 0.20 (0.06,0.64) |  |
| **Smoking status** |  |  |  |  | **0.8627** |
| Never | 1.00 (ref) | 0.50 (0.31,0.82) | 0.26 (0.11,0.58) | 0.24 (0.08,0.70) |  |
| Ever | 1.00 (ref) | 0.56 (0.40,0.78) | 0.41 (0.25,0.68) | 0.33 (0.16,0.70) |  |
| **Alcohol intake** |  |  |  |  | **0.2922** |
| Healthy | 1.00 (ref) | 0.49 (0.35,0.68) | 0.33 (0.20,0.56) | 0.20 (0.08,0.50) |  |
| Unhealthy | 1.00 (ref) | 0.71 (0.42,1.22) | 0.51 (0.24,1.10) | 0.61 (0.25,1.46) |  |
| **Diet score** |  |  |  |  | **0.4898** |
| < 4 | 1.00 (ref) | 0.62 (0.43,0.91) | 0.43 (0.25,0.75) | 0.37 (0.16,0.87) |  |
| ≥ 4 | 1.00 (ref) | 0.48 (0.32,0.71) | 0.31 (0.16,0.59) | 0.27 (0.12,0.65) |  |
| **Sleep score** |  |  |  |  | **0.1983** |
| < 3 | 1.00 (ref) | 0.70 (0.44,1.12) | 0.48 (0.23,0.98) | 0.82 (0.34,2.00) |  |
| ≥ 3 | 1.00 (ref) | 0.48 (0.34,0.68) | 0.33 (0.19,0.55) | 0.19 (0.08,0.44) |  |
| **History of hypertension** |  |  |  |  | **0.1044** |
| No | 1.00 (ref) | 0.45 (0.29,0.70) | 0.30 (0.16,0.56) | 0.14 (0.04,0.47) |  |
| Yes | 1.00 (ref) | 0.64 (0.45,0.91) | 0.45 (0.25,0.78) | 0.52 (0.26,1.05) |  |

* Hazard ratios were calculated in Cox proportional hazards model after adjusting for age (years), sex (male or female), ethnicity (white or others), education (college/university or others), season at the time of accelerometry recording (spring, summer, autumn, or winter), accelerometer wear duration (days), smoking status (never, former, or current), alcohol intake (g/day), diet score (0 to 7), sleep score (0 to 5), body mass index (kg/m^2^), waist circumference (cm), self-rated health (excellent, good, fair, or poor), long-standing illness, disability or infirmity (yes or no), illness, injury, bereavement, or stress in last 2 years (yes or no), history of cancer or cardiovascular disease (yes or no), history of hypertension (yes or no), and diabetes duration (years). Wald tests were used to obtain the two-sided *p-value*.

HR indicates hazard ratio; CI, confidence interval; LPA, light-intensity physical activity; MPA, moderate-intensity physical activity; VPA, vigorous-intensity physical activity; MVPA, moderate-to-vigorous-intensity physical activity.

**Supplementary Table 15. Association of physical activity with all-cause and cause-specific mortality risk, excluding patients with poor self-rated health (n=3527)**.

| **Outcomes** | **LPA (minutes/week)** | | | | ***p-value* for trend** |
| --- | --- | --- | --- | --- | --- |
|  | **< 1750** | **1750-2099** | **2100-2449** | **≥ 2450** |  |
| **All-cause mortality** |  |  |  |  |  |
| No. of cases | 126 | 90 | 36 | 23 |  |
| HR (95% CI) | 1.00 (ref) | 0.94 (0.71,1.24) | 0.58 (0.40,0.84) | 0.70 (0.44,1.10) | =0.009 |
| **Cancer mortality** |  |  |  |  |  |
| No. of cases | 55 | 48 | 18 | 15 |  |
| HR (95% CI) | 1.00 (ref) | 1.13 (0.76,1.68) | 0.64 (0.37,1.09) | 0.97 (0.54,1.76) | =0.406 |
| **Cardiovascular disease mortality** |  |  |  |  |  |
| No. of cases | 36 | 17 | 10 | 2 |  |
| HR (95% CI) | 1.00 (ref) | 0.64 (0.35,1.15) | 0.61 (0.30,1.26) | 0.24 (0.06,1.03) | =0.018 |
|  | **MPA (minutes/week)** | | | | ***p-value* for trend** |
|  | **< 150** | **150-299** | **300-449** | **≥ 450** |  |
| **All-cause mortality** |  |  |  |  |  |
| No. of cases | 88 | 104 | 56 | 27 |  |
| HR (95% CI) | 1.00 (ref) | 0.62 (0.46,0.83) | 0.44 (0.31,0.63) | 0.25 (0.16,0.40) | <0.001 |
| **Cancer mortality** |  |  |  |  |  |
| No. of cases | 32 | 50 | 37 | 17 |  |
| HR (95% CI) | 1.00 (ref) | 0.79 (0.50,1.25) | 0.76 (0.46,1.26) | 0.39 (0.21,0.75) | =0.005 |
| **Cardiovascular disease mortality** |  |  |  |  |  |
| No. of cases | 23 | 27 | 10 | 5 |  |
| HR (95% CI) | 1.00 (ref) | 0.67 (0.37,1.20) | 0.35 (0.16,0.76) | 0.24 (0.09,0.68) | =0.002 |
|  | **VPA (minutes/week)** | | | | ***p-value* for trend** |
|  | **0** | **1-14** | **15-30** | **≥ 30** |  |
| **All-cause mortality** |  |  |  |  |  |
| No. of cases | 140 | 77 | 41 | 17 |  |
| HR (95% CI) | 1.00 (ref) | 0.64 (0.48,0.85) | 0.53 (0.37,0.77) | 0.45 (0.27,0.77) | <0.001 |
| **Cancer mortality** |  |  |  |  |  |
| No. of cases | 59 | 35 | 30 | 12 |  |
| HR (95% CI) | 1.00 (ref) | 0.65 (0.42,0.99) | 0.86 (0.54,1.38) | 0.68 (0.35,1.31) | =0.332 |
| **Cardiovascular disease mortality** |  |  |  |  |  |
| No. of cases | 34 | 25 | 4 | 2 |  |
| HR (95% CI) | 1.00 (ref) | 0.97 (0.56,1.67) | 0.24 (0.08,0.70) | 0.24 (0.06,1.05) | =0.008 |
|  | **MVPA (minutes/week)** | | | | ***p-value* for trend** |
|  | **< 275** | **275-449** | **450-624** | **≥ 625** |  |
| **All-cause mortality** |  |  |  |  |  |
| No. of cases | 177 | 63 | 25 | 10 |  |
| HR (95% CI) | 1.00 (ref) | 0.56 (0.41,0.75) | 0.39 (0.25,0.61) | 0.29 (0.15,0.57) | <0.001 |
| **Cancer mortality** |  |  |  |  |  |
| No. of cases | 75 | 39 | 17 | 5 |  |
| HR (95% CI) | 1.00 (ref) | 0.77 (0.51,1.15) | 0.59 (0.34,1.02) | 0.29 (0.11,0.74) | =0.002 |
| **Cardiovascular disease mortality** |  |  |  |  |  |
| No. of cases | 47 | 12 | 5 | 1 |  |
| HR (95% CI) | 1.00 (ref) | 0.43 (0.22,0.83) | 0.35 (0.13,0.91) | 0.14 (0.02,1.08) | =0.001 |

Hazard ratios were calculated in Cox proportional hazards model after adjusting for age (years), sex (male or female), ethnicity (white or others), education (college/university or others), season at the time of accelerometry recording (spring, summer, autumn, or winter), accelerometer wear duration (days), smoking status (never, former, or current), alcohol intake (g/day), diet score (0 to 7), sleep score (0 to 5), body mass index (kg/m^2^), waist circumference (cm), self-rated health (excellent, good, fair, or poor), long-standing illness, disability or infirmity (yes or no), illness, injury, bereavement, or stress in last 2 years (yes or no), history of cancer or cardiovascular disease (yes or no), history of hypertension (yes or no), and diabetes duration (years). Wald tests were used to obtain the two-sided *p-value*.

HR indicates hazard ratio; CI, confidence interval; LPA, light-intensity physical activity; MPA, moderate-intensity physical activity; VPA, vigorous-intensity physical activity; MVPA, moderate-to-vigorous-intensity physical activity.

**Supplementary Table 16. Association between physical activity and all-cause mortality risk stratified by diabetes severity status (n = 3766)** ***.**

| **No. of diabetes severity factors ^※^** | **No. of participants** | **LPA (minutes/week)** | | | | ***p-value* for interaction** |
| --- | --- | --- | --- | --- | --- | --- |
|  |  | **< 1750** | **1750-2099** | **2100-2449** | **≥ 2450** |  |
| None | 1666 | 1.00 (ref) | 0.68 (0.43,1.07) | 0.44 (0.25,0.79) | 0.56 (0.26,1.20) | 0.7373 |
| One | 1210 | 1.00 (ref) | 1.21 (0.79,1.86) | 0.83 (0.46,1.48) | 0.66 (0.31,1.43) |  |
| Two or three | 890 | 1.00 (ref) | 0.91 (0.56,1.48) | 0.44 (0.22,0.86) | 0.58 (0.24,1.41) |  |
|  |  | **MPA (minutes/week)** | | | | ***p-value* for interaction** |
|  |  | **< 150** | **150-299** | **300-449** | **≥ 450** |  |
| None | 1666 | 1.00 (ref) | 0.54 (0.34,0.85) | 0.32 (0.18,0.57) | 0.19 (0.09,0.41) | 0.7208 |
| One | 1210 | 1.00 (ref) | 0.56 (0.35,0.89) | 0.55 (0.32,0.95) | 0.28 (0.14,0.57) |  |
| Two or three | 890 | 1.00 (ref) | 0.77 (0.47,1.26) | 0.41 (0.21,0.80) | 0.20 (0.07,0.57) |  |
|  |  | **VPA (minutes/week)** | | | | ***p-value* for interaction** |
|  |  | **0** | **1-14** | **15-30** | **≥ 30** |  |
| None | 1666 | 1.00 (ref) | 0.63 (0.40,0.99) | 0.46 (0.25,0.83) | 0.69 (0.32,1.48) | 0.3371 |
| One | 1210 | 1.00 (ref) | 0.50 (0.32,0.79) | 0.66 (0.38,1.14) | 0.32 (0.13,0.84) |  |
| Two or three | 890 | 1.00 (ref) | 0.87 (0.52,1.44) | 0.59 (0.30,1.15) | 0.38 (0.11,1.28) |  |
|  |  | **MVPA (minutes/week)** | | | | ***p-value* for interaction** |
|  |  | **< 275** | **275-449** | **450-624** | **≥ 625** |  |
| None | 1666 | 1.00 (ref) | 0.49 (0.31,0.79) | 0.20 (0.08,0.46) | 0.45 (0.18,1.09) | 0.3040 |
| One | 1210 | 1.00 (ref) | 0.71 (0.45,1.12) | 0.59 (0.32,1.07) | 0.18 (0.04,0.77) |  |
| Two or three | 890 | 1.00 (ref) | 0.43 (0.24,0.76) | 0.33 (0.13,0.86) | 0.19 (0.04,0.83) |  |

* Participants with missing information on glycated hemoglobin level were excluded.

**^※^** Includes three factors: glycated hemoglobin level ≥53mmol/mol (7.0%), diabetes duration ≥10 years, and insulin medication use.

Hazard ratios were calculated in Cox proportional hazards model after adjusting for age (years), sex (male or female), ethnicity (white or others), education (college/university or others), season at the time of accelerometry recording (spring, summer, autumn, or winter), accelerometer wear duration (days), smoking status (never, former, or current), alcohol intake (g/day), diet score (0 to 7), sleep score (0 to 5), body mass index (kg/m^2^), waist circumference (cm), self-rated health (excellent, good, fair, or poor), long-standing illness, disability or infirmity (yes or no), illness, injury, bereavement, or stress in last 2 years (yes or no), history of cancer or cardiovascular disease (yes or no), history of hypertension (yes or no), and diabetes duration (years). Wald tests were used to obtain the two-sided *p-value*.

HR indicates hazard ratio; CI, confidence interval; LPA, light-intensity physical activity; MPA, moderate-intensity physical activity; VPA, vigorous-intensity physical activity; MVPA, moderate-to-vigorous-intensity physical activity.

**Supplementary Table 17. Association of physical activity with all-cause mortality risk after adjusting for diabetes severity factors (n = 3766).**

| **Exposures** | **HR (95% CI)** |
| --- | --- |
| **LPA (minutes/week)** |  |
| < 1750 | 1.00 (ref) |
| 1750-2099 | 0.92 (0.71,1.19) |
| 2100-2449 | 0.58 (0.41,0.82) |
| ≥ 2450 | 0.58 (0.37,0.92) |
| **MPA (minutes/week)** |  |
| < 150 | 1.00 (ref) |
| 150-299 | 0.61 (0.46,0.79) |
| 300-449 | 0.42 (0.30,0.59) |
| ≥ 450 | 0.23 (0.15,0.37) |
| **VPA (minutes/week)** |  |
| 0 | 1.00 (ref) |
| 1-14 | 0.61 (0.47,0.80) |
| 15-29 | 0.55 (0.39,0.77) |
| ≥ 30 | 0.43 (0.25,0.72) |
| **MVPA (minutes/week)** |  |
| < 275 | 1.00 (ref) |
| 275-449 | 0.55 (0.41,0.72) |
| 450-624 | 0.37 (0.24,0.57) |
| ≥ 625 | 0.28 (0.14,0.54) |

Hazard ratios were calculated in Cox proportional hazards model after adjusting for age (years), sex (male or female), ethnicity (white or others), education (college/university or others), season at the time of accelerometry recording (spring, summer, autumn, or winter), accelerometer wear duration (days), smoking status (never, former, or current), alcohol intake (g/day), diet score (0 to 7), sleep score (0 to 5), body mass index (kg/m^2^), waist circumference (cm), self-rated health (excellent, good, fair, or poor), long-standing illness, disability or infirmity (yes or no), illness, injury, bereavement, or stress in last 2 years (yes or no), history of cancer or cardiovascular disease (yes or no), history of hypertension (yes or no), and diabetes duration (years).

HR indicates hazard ratio; CI, confidence interval; LPA, light-intensity physical activity; MPA, moderate-intensity physical activity; VPA, vigorous-intensity physical activity; MVPA, moderate-to-vigorous-intensity physical activity.

**Supplementary Table 18. Association between physical activity and all-cause and cause-specific mortality risk, additionally adjusted for diabetes related disease.**

| **Exposures** | **HR (95% CI)** | | |
| --- | --- | --- | --- |
|  | **All-cause** | **Cancer** | **Cardiovascular disease** |
| **LPA (minutes/week)** |  |  |  |
| < 1750 | 1.00 (ref) | 1.00 (ref) | 1.00 (ref) |
| 1750-2099 | 0.91 (0.71,1.17) | 1.09 (0.75,1.57) | 0.64 (0.37,1.10) |
| 2100-2449 | 0.62 (0.44,0.86) | 0.57 (0.34,0.96) | 0.73 (0.40,1.35) |
| ≥ 2450 | 0.63 (0.41,0.97) | 0.81 (0.45,1.44) | 0.31 (0.10,1.02) |
| **MPA (minutes/week)** |  |  |  |
| < 150 | 1.00 (ref) | 1.00 (ref) | 1.00 (ref) |
| 150-299 | 0.62 (0.48,0.81) | 0.73 (0.48,1.09) | 0.81 (0.48,1.35) |
| 300-449 | 0.42 (0.30,0.58) | 0.69 (0.44,1.10) | 0.37 (0.18,0.77) |
| ≥ 450 | 0.25 (0.16,0.38) | 0.36 (0.20,0.66) | 0.32 (0.13,0.78) |
| **VPA (minutes/week)** |  |  |  |
| 0 | 1.00 (ref) | 1.00 (ref) | 1.00 (ref) |
| 1-14 | 0.59 (0.46,0.77) | 0.58 (0.39,0.86) | 0.99 (0.61,1.61) |
| 15-29 | 0.54 (0.38,0.75) | 0.79 (0.51,1.23) | 0.39 (0.17,0.88) |
| ≥ 30 | 0.45 (0.27,0.73) | 0.60 (0.31,1.14) | 0.35 (0.10,1.17) |
| **MVPA (minutes/week)** |  |  |  |
| < 275 | 1.00 (ref) | 1.00 (ref) | 1.00 (ref) |
| 275-449 | 0.55 (0.42,0.72) | 0.72 (0.49,1.05) | 0.50 (0.28,0.89) |
| 450-624 | 0.37 (0.24,0.56) | 0.56 (0.33,0.94) | 0.32 (0.13,0.83) |
| ≥ 625 | 0.32 (0.17,0.58) | 0.27 (0.10,0.68) | 0.38 (0.11,1.28) |

Hazard ratios were calculated in Cox proportional hazards model after adjusting for age (years), sex (male or female), ethnicity (white or others), education (college/university or others), season at the time of accelerometry recording (spring, summer, autumn, or winter), accelerometer wear duration (days), smoking status (never, former, or current), alcohol intake (g/day), diet score (0 to 7), sleep score (0 to 5), body mass index (kg/m^2^), waist circumference (cm), self-rated health (excellent, good, fair, or poor), long-standing illness, disability or infirmity (yes or no), illness, injury, bereavement, or stress in last 2 years (yes or no), history of cancer or cardiovascular disease (yes or no), history of hypertension (yes or no), and diabetes duration (years).

HR indicates hazard ratio; CI, confidence interval; LPA, light-intensity physical activity; MPA, moderate-intensity physical activity; VPA, vigorous-intensity physical activity; MVPA, moderate-to-vigorous-intensity physical activity.

**Supple**mentary Table 19. Association between physical activity and all-cause and cause-specific mortality risk, mutually adjusted for different PA intensities.

| **Exposures** | **HR (95% CI)** | | |
| --- | --- | --- | --- |
|  | **All-cause** | **Cancer** | **Cardiovascular disease** |
| **LPA (minutes/week)** |  |  |  |
| < 1750 | 1.00 (ref) | 1.00 (ref) | 1.00 (ref) |
| 1750-2099 | 1.17 (0.90,1.52) | 1.24 (0.84,1.82) | 0.78 (0.44,1.36) |
| 2100-2449 | 0.91 (0.64,1.30) | 0.71 (0.41,1.23) | 1.06 (0.55,2.04) |
| ≥ 2450 | 1.04 (0.66,1.64) | 1.14 (0.61,2.10) | 0.44 (0.13,1.50) |
| ***p-value* for trend** | =0.991 | =0.833 | =0.365 |
| **MPA (minutes/week)** |  |  |  |
| < 150 | 1.00 (ref) | 1.00 (ref) | 1.00 (ref) |
| 150-299 | 0.65 (0.49,0.87) | 0.79 (0.50,1.23) | 0.84 (0.48,1.48) |
| 300-449 | 0.46 (0.31,0.68) | 0.76 (0.43,1.34) | 0.43 (0.19,1.00) |
| ≥ 450 | 0.27 (0.16,0.47) | 0.38 (0.18,0.80) | 0.48 (0.17,1.40) |
| ***p-value* for trend** | <0.001 | =0.014 | =0.080 |
| **VPA (minutes/week)** |  |  |  |
| 0 | 1.00 (ref) | 1.00 (ref) | 1.00 (ref) |
| 1-14 | 0.79 (0.59,1.05) | 0.66 (0.43,1.02) | 1.28 (0.75,2.18) |
| 15-29 | 0.91 (0.62,1.34) | 1.12 (0.67,1.88) | 0.60 (0.24,1.49) |
| ≥ 30 | 0.86 (0.50,1.51) | 0.94 (0.45,1.94) | 0.58 (0.15,2.14) |
| ***p-value* for trend** | =0.588 | =0.888 | =0.338 |
| **MVPA (minutes/week)** |  |  |  |
| < 275 | 1.00 (ref) | 1.00 (ref) | 1.00 (ref) |
| 275-449 | 0.56 (0.42,0.75) | 0.72 (0.48,1.07) | 0.55 (0.30,0.99) |
| 450-624 | 0.38 (0.25,0.59) | 0.58 (0.33,1.00) | 0.36 (0.14,0.93) |
| ≥ 625 | 0.34 (0.18,0.63) | 0.28 (0.11,0.73) | 0.44 (0.13,1.54) |
| ***p-value* for trend** | <0.001 | =0.002 | =0.016 |

Hazard ratios were calculated in Cox proportional hazards model after adjusting for age (years), sex (male or female), ethnicity (white or others), education (college/university or others), season at the time of accelerometry recording (spring, summer, autumn, or winter), accelerometer wear duration (days), smoking status (never, former, or current), alcohol intake (g/day), diet score (0 to 7), sleep score (0 to 5), body mass index (kg/m^2^), waist circumference (cm), self-rated health (excellent, good, fair, or poor), long-standing illness, disability or infirmity (yes or no), illness, injury, bereavement, or stress in last 2 years (yes or no), history of cancer or cardiovascular disease (yes or no), history of hypertension (yes or no), and diabetes duration (years). Wald tests were used to obtain the two-sided *p-value*.

LPA, MPA, and VPA were mutually adjusted for in the models.

HR indicates hazard ratio; CI, confidence interval; LPA, light-intensity physical activity; MPA, moderate-intensity physical activity; VPA, vigorous-intensity physical activity; MVPA, moderate-to-vigorous-intensity physical activity.

**Supplementary Table 20. Association of physical activity with all-cause and cause-specific mortality risk using multiple imputations with chained equations (n = 4118)**.

| **Outcomes** | **LPA (minutes/week)** | | | |
| --- | --- | --- | --- | --- |
|  | **< 1750** | **1750-2099** | **2100-2449** | **≥ 2450** |
| **All-cause mortality** |  |  |  |  |
| No. of cases | 173 | 109 | 47 | 25 |
| HR (95% CI) | 1.00 (ref) | 0.96 (0.75,1.23) | 0.62 (0.45,0.87) | 0.61 (0.39,0.93) |
| **Cancer mortality** |  |  |  |  |
| No. of cases | 72 | 58 | 19 | 15 |
| HR (95% CI) | 1.00 (ref) | 1.16 (0.81,1.66) | 0.57 (0.34,0.95) | 0.78 (0.44,1.39) |
| **Cardiovascular disease mortality** |  |  |  |  |
| No. of cases | 50 | 22 | 15 | 3 |
| HR (95% CI) | 1.00 (ref) | 0.7 (0.41,1.18) | 0.74 (0.40,1.37) | 0.3 (0.09,1.00) |
|  | **MPA (minutes/week)** | | | |
|  | **< 150** | **150-299** | **300-449** | **≥ 450** |
| **All-cause mortality** |  |  |  |  |
| No. of cases | 123 | 134 | 65 | 32 |
| HR (95% CI) | 1.00 (ref) | 0.65 (0.50,0.84) | 0.45 (0.33,0.62) | 0.27 (0.18,0.41) |
| **Cancer mortality** |  |  |  |  |
| No. of cases | 44 | 58 | 43 | 19 |
| HR (95% CI) | 1.00 (ref) | 0.75 (0.50,1.13) | 0.77 (0.49,1.21) | 0.41 (0.22,0.73) |
| **Cardiovascular disease mortality** |  |  |  |  |
| No. of cases | 31 | 40 | 12 | 7 |
| HR (95% CI) | 1.00 (ref) | 0.83 (0.50,1.36) | 0.38 (0.19,0.78) | 0.3 (0.12,0.75) |
|  | **VPA (minutes/week)** | | | |
|  | **0** | **1-14** | **15-29** | **≥ 30** |
| **All-cause mortality** |  |  |  |  |
| No. of cases | 190 | 92 | 52 | 20 |
| HR (95% CI) | 1.00 (ref) | 0.61 (0.47,0.78) | 0.57 (0.41,0.79) | 0.46 (0.28,0.75) |
| **Cancer mortality** |  |  |  |  |
| No. of cases | 78 | 39 | 35 | 12 |
| HR (95% CI) | 1.00 (ref) | 0.59 (0.39,0.88) | 0.88 (0.57,1.36) | 0.6 (0.31,1.15) |
| **Cardiovascular disease mortality** |  |  |  |  |
| No. of cases | 47 | 33 | 7 | 3 |
| HR (95% CI) | 1.00 (ref) | 0.97 (0.60,1.56) | 0.34 (0.15,0.80) | 0.31 (0.09,1.07) |
|  | **MVPA (minutes/week)** | | | |
|  | **< 275** | **275-449** | **450-624** | **≥ 625** |
| **All-cause mortality** |  |  |  |  |
| No. of cases | 237 | 77 | 28 | 12 |
| HR (95% CI) | 1.00 (ref) | 0.57 (0.44,0.75) | 0.39 (0.26,0.59) | 0.31 (0.17,0.57) |
| **Cancer mortality** |  |  |  |  |
| No. of cases | 95 | 45 | 19 | 5 |
| HR (95% CI) | 1.00 (ref) | 0.77 (0.53,1.12) | 0.6 (0.36,1.02) | 0.28 (0.11,0.72) |
| **Cardiovascular disease mortality** |  |  |  |  |
| No. of cases | 65 | 17 | 5 | 3 |
| HR (95% CI) | 1.00 (ref) | 0.50 (0.28,0.88) | 0.29 (0.11,0.76) | 0.36 (0.10,1.24) |

Hazard ratios were calculated in Cox proportional hazards model after adjusting for age (years), sex (male or female), ethnicity (white or others), education (college/university or others), season at the time of accelerometry recording (spring, summer, autumn, or winter), accelerometer wear duration (days), smoking status (never, former, or current), alcohol intake (g/day), diet score (0 to 7), sleep score (0 to 5), body mass index (kg/m^2^), waist circumference (cm), self-rated health (excellent, good, fair, or poor), long-standing illness, disability or infirmity (yes or no), illness, injury, bereavement, or stress in last 2 years (yes or no), history of cancer or cardiovascular disease (yes or no), history of hypertension (yes or no), and diabetes duration (years).

HR indicates hazard ratio; CI, confidence interval; LPA, light-intensity physical activity; MPA, moderate-intensity physical activity; VPA, vigorous-intensity physical activity; MVPA, moderate-to-vigorous-intensity physical activity.

**Supplementary Table 21. Association of physical activity with cancer and cardiovascular disease mortality risk using Fine & Gray models for competing risk (n = 4003).**

| **Outcomes** | **LPA (minutes/week)** | | | |
| --- | --- | --- | --- | --- |
|  | **< 1750** | **1750-2099** | **2100-2449** | **≥ 2450** |
| **Cancer** **mortality** |  |  |  |  |
| HR (95% CI) | 1.00 (ref) | 1.10 (0.77,1.58) | 0.58 (0.35,0.96) | 0.81 (0.46,1.43) |
| **Cardiovascular disease mortality** |  |  |  |  |
| HR (95% CI) | 1.00 (ref) | 0.64 (0.37,1.12) | 0.77 (0.42,1.41) | 0.32 (0.09,1.08) |
|  | **MPA (minutes/week)** | | | |
|  | **< 150** | **150-299** | **300-449** | **≥ 450** |
| **Cancer** **mortality** |  |  |  |  |
| HR (95% CI) | 1.00 (ref) | 0.73 (0.48,1.11) | 0.71 (0.45,1.11) | 0.37 (0.20,0.68) |
| **Cardiovascular disease mortality** |  |  |  |  |
| HR (95% CI) | 1.00 (ref) | 0.85 (0.50,1.44) | 0.39 (0.18,0.81) | 0.34 (0.14,0.83) |
|  | **VPA (minutes/week)** | | | |
|  | **0** | **1-14** | **15-29** | **≥ 30** |
| **Cancer** **mortality** |  |  |  |  |
| HR (95% CI) | 1.00 (ref) | 0.58 (0.39,0.86) | 0.81 (0.53,1.23) | 0.61 (0.31,1.18) |
| **Cardiovascular disease mortality** |  |  |  |  |
| HR (95% CI) | 1.00 (ref) | 1.03 (0.63,1.69) | 0.40 (0.17,0.94) | 0.37 (0.11,1.19) |
|  | **MVPA (minutes/week)** | | | |
|  | **< 275** | **275-449** | **450-624** | **≥ 625** |
| **Cancer** **mortality** |  |  |  |  |
| HR (95% CI) | 1.00 (ref) | 0.73 (0.50,1.07) | 0.57 (0.34,0.95) | 0.27 (0.11,0.69) |
| **Cardiovascular disease mortality** |  |  |  |  |
| HR (95% CI) | 1.00 (ref) | 0.51 (0.29,0.91) | 0.34 (0.13,0.84) | 0.40 (0.12,1.32) |

Hazard ratios were calculated in Cox proportional hazards model after adjusting for age (years), sex (male or female), ethnicity (white or others), education (college/university or others), season at the time of accelerometry recording (spring, summer, autumn, or winter), accelerometer wear duration (days), smoking status (never, former, or current), alcohol intake (g/day), diet score (0 to 7), sleep score (0 to 5), body mass index (kg/m^2^), waist circumference (cm), self-rated health (excellent, good, fair, or poor), long-standing illness, disability or infirmity (yes or no), illness, injury, bereavement, or stress in last 2 years (yes or no), history of cancer or cardiovascular disease (yes or no), history of hypertension (yes or no), and diabetes duration (years).

HR indicates hazard ratio; CI, confidence interval; LPA, light-intensity physical activity; MPA, moderate-intensity physical activity; VPA, vigorous-intensity physical activity; MVPA, moderate-to-vigorous-intensity physical activity.

**Supplementary Table 22. Association between** physical activity and all-cause and cause-specific mortality risk converting the duration data into MET-minutes/week.

| **Exposures** | **HR (95% CI) for every 600 MET-minutes/week** **increase** | | |
| --- | --- | --- | --- |
|  | **All-cause mortality** | **Cancer mortality** | **CVD mortality** |
| Total physical activity | 0.89 (0.86,0.92) | 0.91 (0.87,0.96) | 0.90 (0.84,0.97) |

Hazard ratios were calculated in Cox proportional hazards model after adjusting for age (years), sex (male or female), ethnicity (white or others), education (college/university or others), season at the time of accelerometry recording (spring, summer, autumn, or winter), accelerometer wear duration (days), smoking status (never, former, or current), alcohol intake (g/day), diet score (0 to 7), sleep score (0 to 5), body mass index (kg/m^2^), waist circumference (cm), self-rated health (excellent, good, fair, or poor), long-standing illness, disability or infirmity (yes or no), illness, injury, bereavement, or stress in last 2 years (yes or no), history of cancer or cardiovascular disease (yes or no), history of hypertension (yes or no), and diabetes duration (years).

HR indicates hazard ratio; CI, confidence interval; CVD, cardiovascular disease; MET, metabolic equivalent of task.

Supplementary Table 23. Association between physical activity and all-cause and cause-specific mortality risk grouping participants into two groups.

| **Exposures** | **HR (95% CI)** | | |
| --- | --- | --- | --- |
|  | **All-cause** | **Cancer** | **Cardiovascular disease** |
| Not meeting the recommendations of MPA | 1 (ref) | 1 (ref) | 1 (ref) |
| Meeting the recommendations of MPA | 0.50 (0.39,0.64) | 0.66 (0.45,0.96) | 0.61 (0.37,1.00) |
| Not meeting the recommendations of VPA | 1 (ref) | 1 (ref) | 1 (ref) |
| Meeting the recommendations of VPA | 1.04 (0.46,2.37) | 1.62 (0.65,4.06) | NA |

Hazard ratios were calculated in Cox proportional hazards model after adjusting for age (years), sex (male or female), ethnicity (white or others), education (college/university or others), season at the time of accelerometry recording (spring, summer, autumn, or winter), accelerometer wear duration (days), smoking status (never, former, or current), alcohol intake (g/day), diet score (0 to 7), sleep score (0 to 5), body mass index (kg/m^2^), waist circumference (cm), self-rated health (excellent, good, fair, or poor), long-standing illness, disability or infirmity (yes or no), illness, injury, bereavement, or stress in last 2 years (yes or no), history of cancer or cardiovascular disease (yes or no), history of hypertension (yes or no), and diabetes duration (years).

The guideline’s criterion for recommended is 150 minutes/week for MPA and 75 minutes/week for VPA.

HR indicates hazard ratio; CI, confidence interval; LPA, light-intensity physical activity; MPA, moderate-intensity physical activity; VPA, vigorous-intensity physical activity; MVPA, moderate-to-vigorous-intensity physical activity.

**Supplementary Table 24. Association between physical activity and risk of mortality from cancer and CVD among participants without the corresponding disease at baseline.**

| **Outcomes** | **LPA (minutes/week)** | | | |
| --- | --- | --- | --- | --- |
|  | **< 1750** | **1750-2099** | **2100-2449** | **≥ 2450** |
| **Cancer mortality** |  |  |  |  |
| No. of cases | 49 | 34 | 15 | 15 |
| HR (95% CI) | 1.00 (ref) | 0.96 (0.61,1.50) | 0.64 (0.36,1.16) | 1.05 (0.57,1.92) |
| **Cardiovascular disease mortality** |  |  |  |  |
| No. of cases | 21 | 14 | 7 | 2 |
| HR (95% CI) | 1.00 (ref) | 0.94 (0.47,1.89) | 0.69 (0.29,1.65) | 0.44 (0.10,1.91) |
|  | **MPA (minutes/week)** | | | |
|  | **< 150** | **150-299** | **300-449** | **≥ 450** |
| **Cancer mortality** |  |  |  |  |
| No. of cases | 30 | 40 | 27 | 16 |
| HR (95% CI) | 1.00 (ref) | 0.73 (0.45,1.20) | 0.67 (0.38,1.17) | 0.45 (0.23,0.89) |
| **Cardiovascular disease mortality** |  |  |  |  |
| No. of cases | 15 | 16 | 9 | 4 |
| HR (95% CI) | 1.00 (ref) | 0.59 (0.28,1.24) | 0.48 (0.20,1.17) | 0.28 (0.08,0.92) |
|  | **VPA (minutes/week)** | | | |
|  | **0** | **1-14** | **15-30** | **≥ 30** |
| **Cancer mortality** |  |  |  |  |
| No. of cases | 49 | 27 | 26 | 11 |
| HR (95% CI) | 1.00 (ref) | 0.62 (0.38,1.00) | 1.08 (0.65,1.81) | 0.84 (0.42,1.69) |
| **Cardiovascular disease mortality** |  |  |  |  |
| No. of cases | 20 | 20 | 3 | 1 |
| HR (95% CI) | 1.00 (ref) | 1.32 (0.69,2.55) | 0.34 (0.10,1.20) | 0.23 (0.03,1.76) |
|  | **MVPA (minutes/week)** | | | |
|  | **< 275** | **275-449** | **450-624** | **≥ 625** |
| **Cancer mortality** |  |  |  |  |
| No. of cases | 64 | 28 | 16 | 5 |
| HR (95% CI) | 1.00 (ref) | 0.68 (0.43,1.08) | 0.73 (0.41,1.31) | 0.37 (0.14,0.95) |
| **Cardiovascular disease mortality** |  |  |  |  |
| No. of cases | 27 | 12 | 4 | 1 |
| HR (95% CI) | 1.00 (ref) | 0.83 (0.40,1.70) | 0.54 (0.18,1.63) | 0.25 (0.03,1.96) |

Hazard ratios were calculated in Cox proportional hazards model after adjusting for age (years), sex (male or female), ethnicity (white or others), education (college/university or others), season at the time of accelerometry recording (spring, summer, autumn, or winter), accelerometer wear duration (days), smoking status (never, former, or current), alcohol intake (g/day), diet score (0 to 7), sleep score (0 to 5), body mass index (kg/m^2^), waist circumference (cm), self-rated health (excellent, good, fair, or poor), long-standing illness, disability or infirmity (yes or no), illness, injury, bereavement, or stress in last 2 years (yes or no), history of cancer or cardiovascular disease (yes or no), history of hypertension (yes or no), and diabetes duration (years).

HR indicates hazard ratio; CI, confidence interval; LPA, light-intensity physical activity; MPA, moderate-intensity physical activity; VPA, vigorous-intensity physical activity; MVPA, moderate-to-vigorous-intensity physical activity.

**Supplementary Table 25. Association of physical activity with all-cause and cause-specific mortality risk, excluding deaths that occurred within the first 2 or 4 years of follow-up**.

|  | **All-cause mortality** | **Cancer mortality** | **Cardiovascular**  **disease mortality** |
| --- | --- | --- | --- |
| **Excluding deaths that occurred within the first 2 years of follow-up (n = 3924)** | | | |
| **LPA (minutes/week)** |  |  |  |
| < 1750 | 1.00 (ref) | 1.00 (ref) | 1.00 (ref) |
| 1750-2099 | 0.91 (0.68,1.22) | 1.07 (0.69,1.67) | 0.68 (0.36,1.27) |
| 2100-2449 | 0.72 (0.50,1.03) | 0.76 (0.43,1.33) | 0.77 (0.39,1.55) |
| ≥2450 | 0.62 (0.38,1.03) | 0.95 (0.50,1.84) | 0.25 (0.06,1.05) |
| **MPA (minutes/week)** |  |  |  |
| < 150 | 1.00 (ref) | 1.00 (ref) | 1.00 (ref) |
| 150-299 | 0.62 (0.46,0.84) | 0.95 (0.57,1.56) | 0.72 (0.40,1.31) |
| 300-449 | 0.45 (0.31,0.65) | 0.94 (0.54,1.65) | 0.43 (0.20,0.95) |
| ≥450 | 0.27 (0.17,0.44) | 0.53 (0.26,1.07) | 0.35 (0.13,0.94) |
| **VPA (minutes/week)** |  |  |  |
| 0 | 1.00 (ref) | 1.00 (ref) | 1.00 (ref) |
| 1-14 | 0.61 (0.45,0.83) | 0.63 (0.39,1.01) | 0.95 (0.55,1.65) |
| 15-29 | 0.61 (0.42,0.89) | 1.06 (0.64,1.77) | 0.35 (0.13,0.92) |
| ≥30 | 0.50 (0.28,0.87) | 0.85 (0.41,1.75) | 0.29 (0.07,1.25) |
| **MVPA (minutes/week)** |  |  |  |
| <275 | 1.00 (ref) | 1.00 (ref) | 1.00 (ref) |
| 275-449 | 0.56 (0.41,0.77) | 0.79 (0.50,1.23) | 0.56 (0.29,1.06) |
| 450-624 | 0.41 (0.25,0.65) | 0.68 (0.37,1.24) | 0.34 (0.12,0.98) |
| ≥625 | 0.41 (0.22,0.77) | 0.44 (0.17,1.13) | 0.55 (0.16,1.90) |
| **Excluding deaths that occurred within the first 4 years of follow-up (n = 3851)** | | | |
| **LPA (minutes/week)** |  |  |  |
| < 1750 | 1.00 (ref) | 1.00 (ref) | 1.00 (ref) |
| 1750-2099 | 0.96 (0.68,1.35) | 1.17 (0.69,1.99) | 0.60 (0.29,1.22) |
| 2100-2449 | 0.76 (0.49,1.17) | 0.80 (0.41,1.59) | 0.66 (0.29,1.47) |
| ≥2450 | 0.66 (0.37,1.18) | 1.07 (0.49,2.32) | 0.15 (0.02,1.12) |
| **MPA (minutes/week)** |  |  |  |
| < 150 | 1.00 (ref) | 1.00 (ref) | 1.00 (ref) |
| 150-299 | 0.71 (0.50,1.02) | 1.31 (0.68,2.53) | 0.67 (0.35,1.31) |
| 300-449 | 0.51 (0.33,0.79) | 1.27 (0.62,2.61) | 0.40 (0.17,0.98) |
| ≥450 | 0.32 (0.18,0.57) | 0.82 (0.35,1.97) | 0.21 (0.06,0.77) |
| **VPA (minutes/week)** |  |  |  |
| 0 | 1.00 (ref) | 1.00 (ref) | 1.00 (ref) |
| 1-14 | 0.78 (0.55,1.10) | 0.81 (0.46,1.44) | 1.28 (0.68,2.41) |
| 15-29 | 0.88 (0.58,1.33) | 1.56 (0.86,2.81) | 0.59 (0.21,1.61) |
| ≥30 | 0.44 (0.21,0.92) | 0.57 (0.19,1.69) | 0.52 (0.12,2.34) |
| **MVPA (minutes/week)** |  |  |  |
| <275 | 1.00 (ref) | 1.00 (ref) | 1.00 (ref) |
| 275-449 | 0.62 (0.43,0.89) | 0.95 (0.56,1.61) | 0.59 (0.29,1.20) |
| 450-624 | 0.36 (0.20,0.65) | 0.60 (0.27,1.32) | 0.22 (0.05,0.94) |
| ≥625 | 0.56 (0.28,1.10) | 0.70 (0.26,1.89) | 0.51 (0.11,2.27) |

Hazard ratios were calculated in Cox proportional hazards model after adjusting for age (years), sex (male or female), ethnicity (white or others), education (college/university or others), season at the time of accelerometry recording (spring, summer, autumn, or winter), accelerometer wear duration (days), smoking status (never, former, or current), alcohol intake (g/day), diet score (0 to 7), sleep score (0 to 5), body mass index (kg/m^2^), waist circumference (cm), self-rated health (excellent, good, fair, or poor), long-standing illness, disability or infirmity (yes or no), illness, injury, bereavement, or stress in last 2 years (yes or no), history of cancer or cardiovascular disease (yes or no), history of hypertension (yes or no), and diabetes duration (years).

HR indicates hazard ratio; CI, confidence interval; LPA, light-intensity physical activity; MPA, moderate-intensity physical activity; VPA, vigorous-intensity physical activity; MVPA, moderate-to-vigorous-intensity physical activity.

**Supplementary Table 26. Association of physical activity with T2D/Obesity-related or -independent cancers mortality risk (n = 4003).**

| **Outcomes** | **LPA (minutes/week)** | | | |
| --- | --- | --- | --- | --- |
|  | **< 1750** | **1750-2099** | **2100-2449** | **≥ 2450** |
| **T2D/Obesity-related 5cancers** **mortality** |  |  |  |  |
| No. of cases | 11 | 8 | 5 | 4 |
| HR (95% CI) | 1.00 (ref) | 1.01 (0.40,2.58) | 0.94 (0.32,2.77) | 1.15 (0.34,3.82) |
| **T2D/Obesity-independent cancers mortality** |  |  |  |  |
| No. of cases | 59 | 46 | 14 | 11 |
| HR (95% CI) | 1.00 (ref) | 1.09 (0.73,1.63) | 0.51 (0.28,0.92) | 0.72 (0.37,1.41) |
|  | **MPA (minutes/week)** | | | |
|  | **< 150** | **150-299** | **300-449** | **≥ 450** |
| **T2D/Obesity-related cancers** **mortality** |  |  |  |  |
| No. of cases | 4 | 13 | 6 | 5 |
| HR (95% CI) | 1.00 (ref) | 2.22 (0.70,7.03) | 1.42 (0.38,5.34) | 1.42 (0.34,5.93) |
| **T2D/Obesity-independent cancers mortality** |  |  |  |  |
| No. of cases | 39 | 44 | 34 | 13 |
| HR (95% CI) | 1.00 (ref) | 0.60 (0.38,0.94) | 0.62 (0.38,1.02) | 0.28 (0.14,0.55) |
|  | **VPA (minutes/week)** | | | |
|  | **0** | **1-14** | **15-29** | **≥ 30** |
| **T2D/Obesity-related cancers** **mortality** |  |  |  |  |
| No. of cases | 14 | 5 | 6 | 3 |
| HR (95% CI) | 1.00 (ref) | 0.46 (0.16,1.33) | 0.97 (0.35,2.71) | 1.15 (0.29,4.51) |
| **T2D/Obesity-independent cancers mortality** |  |  |  |  |
| No. of cases | 62 | 33 | 26 | 9 |
| HR (95% CI) | 1.00 (ref) | 0.60 (0.39,0.93) | 0.77 (0.47,1.26) | 0.52 (0.25,1.10) |
|  | **MVPA (minutes/week)** | | | |
|  | **< 275** | **275-449** | **450-624** | **≥ 625** |
| **T2D/Obesity-related cancers** **mortality** |  |  |  |  |
| No. of cases | 16 | 5 | 5 | 2 |
| HR (95% CI) | 1.00 (ref) | 0.54 (0.19,1.52) | 1.00 (0.34,2.94) | 0.71 (0.14,3.45) |
| **T2D/Obesity-independent cancers mortality** |  |  |  |  |
| No. of cases | 77 | 37 | 13 | 3 |
| HR (95% CI) | 1.00 (ref) | 0.76 (0.50,1.14) | 0.49 (0.26,0.90) | 0.19 (0.06,0.62) |

Hazard ratios were calculated in Cox proportional hazards model after adjusting for age (years), sex (male or female), ethnicity (white or others), education (college/university or others), season at the time of accelerometry recording (spring, summer, autumn, or winter), accelerometer wear duration (days), smoking status (never, former, or current), alcohol intake (g/day), diet score (0 to 7), sleep score (0 to 5), body mass index (kg/m^2^), waist circumference (cm), self-rated health (excellent, good, fair, or poor), long-standing illness, disability or infirmity (yes or no), illness, injury, bereavement, or stress in last 2 years (yes or no), history of cancer or cardiovascular disease (yes or no), history of hypertension (yes or no), and diabetes duration (years).

HR indicates hazard ratio; CI, confidence interval; LPA, light-intensity physical activity; MPA, moderate-intensity physical activity; VPA, vigorous-intensity physical activity; MVPA, moderate-to-vigorous-intensity physical activity.

**Supplementary Table 27. Proportional hazard test for the main model.**

| **Model 3 in the primary analysis** | | **Proportional hazard test for global model** |
| --- | --- | --- |
| Exposures | Outcomes | *p-value* |
| LPA (minutes/week) | All-cause mortality | 0.0591 |
| MPA (minutes/week) | All-cause mortality | 0.0647 |
| VPA (minutes/week) | All-cause mortality | 0.2883 |
| MVPA (minutes/week) | All-cause mortality | 0.2472 |
| LPA (minutes/week) | Cancer-cause mortality | 0.3368 |
| MPA (minutes/week) | Cancer-cause mortality | 0.1733 |
| VPA (minutes/week) | Cancer-cause mortality | 0.2266 |
| MVPA (minutes/week) | Cancer-cause mortality | 0.1514 |
| LPA (minutes/week) | CVD-cause mortality | 0.7962 |
| MPA (minutes/week) | CVD-cause mortality | 0.8448 |
| VPA (minutes/week) | CVD-cause mortality | 0.8931 |
| MVPA (minutes/week) | CVD-cause mortality | 0.8538 |

The proportional hazard assumption for Cox models was checked with Schoenfeld residuals.

**Supplementary Fig. 1. Flowchart of participants included in the analysis.**


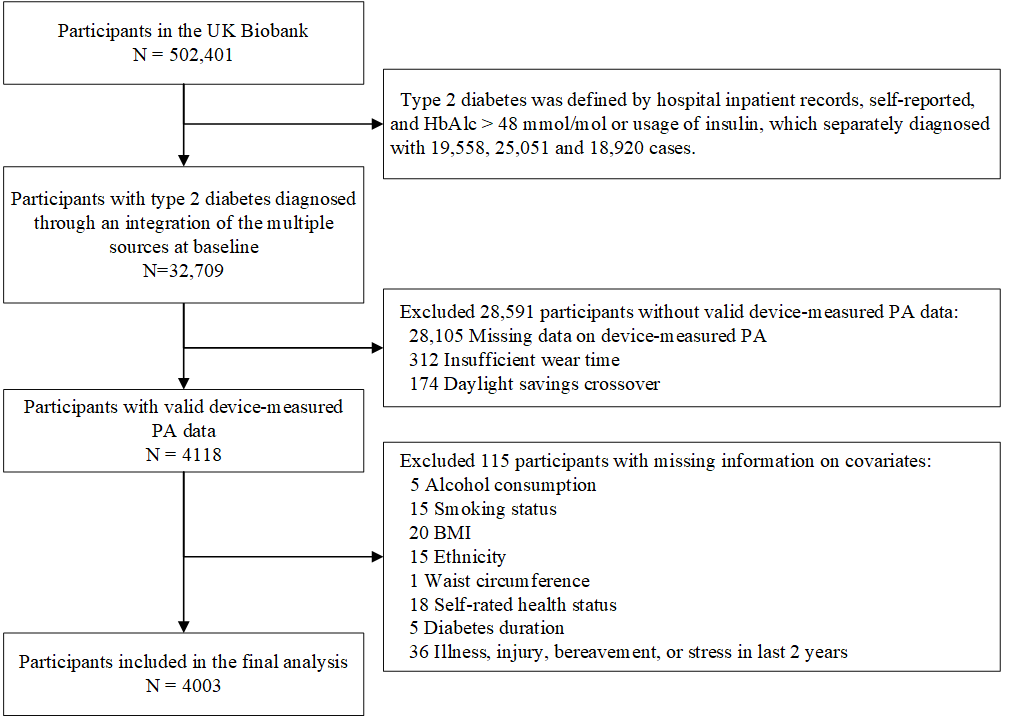


We initially included 32,709 participants who had type 2 diabetes at baseline from 502,401 participants in the UK Biobank. Subsequently, we excluded 28,591 participants without valid accelerometer-measured PA data, which included those who did not attend the accelerometer study, those with insufficient wear time, and those who had daylight saving time shifts during wear period. Among the remaining 4118 participants, 115 were excluded due to missing information on covariates. Thus, 4003 participants with type 2 diabetes were included in the main analysis.

PA, physical activity; BMI, body mass index.

**Supplementary Fig. 2. Dose-response association between physical activity and all-cause and cause-specific mortality, mutually adjusted for PA intensities.**





We utilized diabetic participants (n = 4003) from the UK Biobank with valid accelerometer data in the analyses. **a-d** Dose-response association of LPA, MPA, VPA, and MVPA with all-cause mortality. Bold lines represent HRs, while shaded areas indicate 95% CI. **e-h** Dose-response association of LPA, MPA, VPA, and MVPA with cancer mortality. Bold lines represent HRs, while shaded areas indicate 95% CI. **i-l** Dose-response association of LPA, MPA, VPA, and MVPA with CVD mortality. Bold lines represent HRs, while shaded areas indicate 95% CI.

LPA, MPA, and VPA were mutually adjusted.

All adjusted for age (years), sex (male or female), ethnicity (white or others), education (college/university or others), season at the time of accelerometry recording (spring, summer, autumn, or winter), accelerometer wear duration (days), smoking status (never, former, or current), alcohol intake (g/day), diet score (0 to 7), sleep score (0 to 5), body mass index (kg/m^2^), waist circumference (cm), self-rated health (excellent, good, fair, or poor), long-standing illness, disability or infirmity (yes or no), illness, injury, bereavement, or stress in last 2 years (yes or no), history of cancer or cardiovascular disease (yes or no), history of hypertension (yes or no), and diabetes duration (years). Wald tests were used in the analyses to obtain the two-sided *p-values.*

HR indicates hazard ratio; CI, confidence interval; LPA, light-intensity physical activity; MPA, moderate-intensity physical activity; VPA, vigorous-intensity physical activity; MVPA, moderate-to-vigorous-intensity physical activity.

**Supplementary Fig. 3. Dose-response association between total physical activity and all-cause and cause-specific mortality using the MET-minutes/week as the unit.**


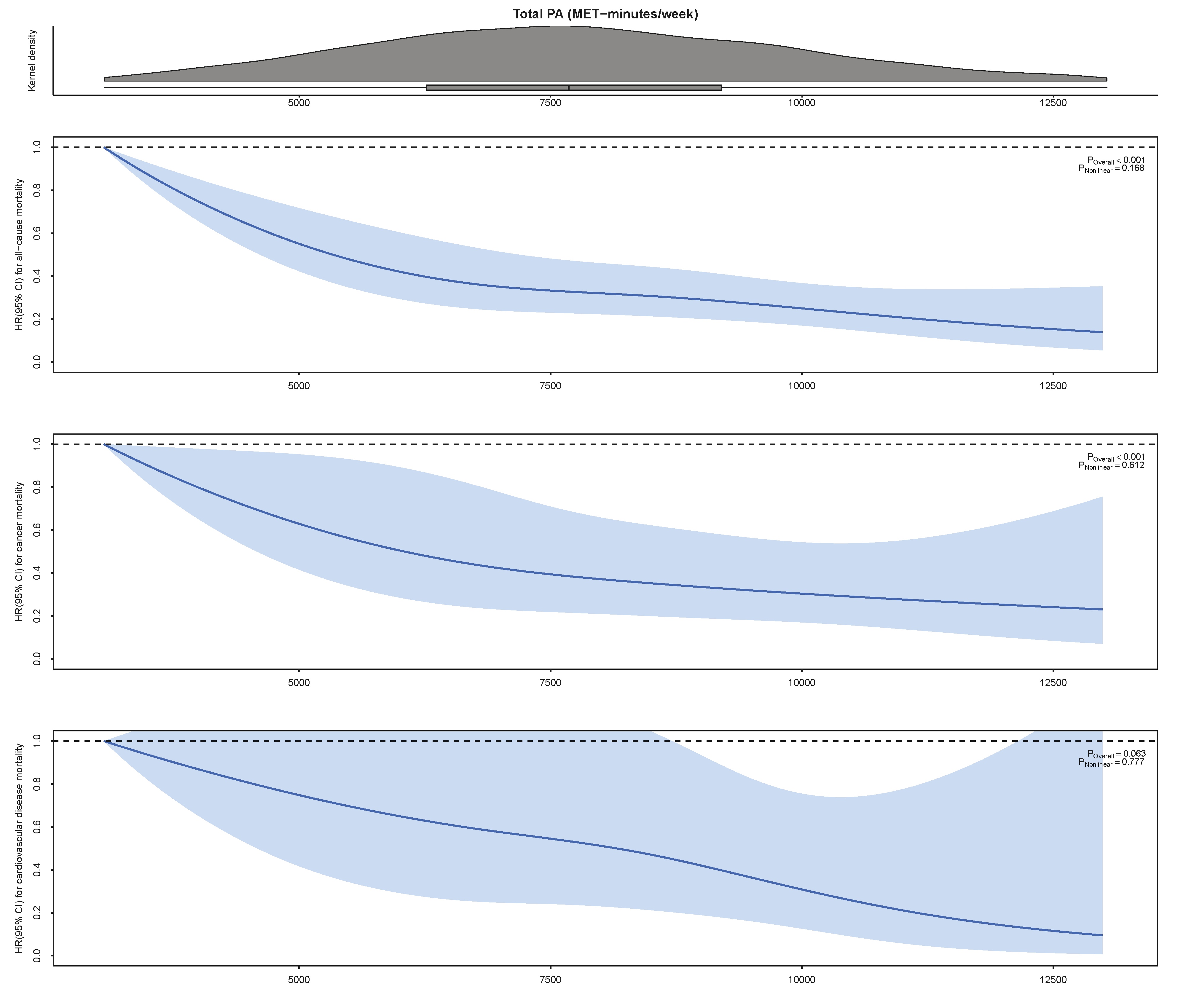


We utilized diabetic participants (n = 4003) from the UK Biobank with valid accelerometer data in the analyses. **a** The gray density histograms depict the distribution of total PA (Met-minutes/week). The data depicted in the box and whisker plots span from the minimum to the maximum values (Met-minutes/week). The lower and upper boundaries of the box correspond to the 25^th^ and 75^th^ percentiles, respectively, and the central vertical lines within the boxes represent the median values. **b** Dose-response association of total PA with all-cause mortality. Bold lines represent HRs, while shaded areas indicate 95% CI. **c** Dose-response association of total PA with cancer mortality. Bold lines represent HRs, while shaded areas indicate 95% CI. **d** Dose-response association of total PA with CVD mortality. Bold lines represent HRs, while shaded areas indicate 95% CI.

All adjusted for age (years), sex (male or female), ethnicity (white or others), education (college/university or others), season at the time of accelerometry recording (spring, summer, autumn, or winter), accelerometer wear duration (days), smoking status (never, former, or current), alcohol intake (g/day), diet score (0 to 7), sleep score (0 to 5), body mass index (kg/m^2^), waist circumference (cm), self-rated health (excellent, good, fair, or poor), long-standing illness, disability or infirmity (yes or no), illness, injury, bereavement, or stress in last 2 years (yes or no), history of cancer or cardiovascular disease (yes or no), history of hypertension (yes or no), and diabetes duration (years). Wald tests were used in the analyses to obtain the two-sided *p-values.*

HR indicates hazard ratio; CI, confidence interval; LPA, light-intensity physical activity; MPA, moderate-intensity physical activity; VPA, vigorous-intensity physical activity; MVPA, moderate-to-vigorous-intensity physical activity.

**Supplementary Fig. 4. The directed acyclic graph to guide covariate selection.**


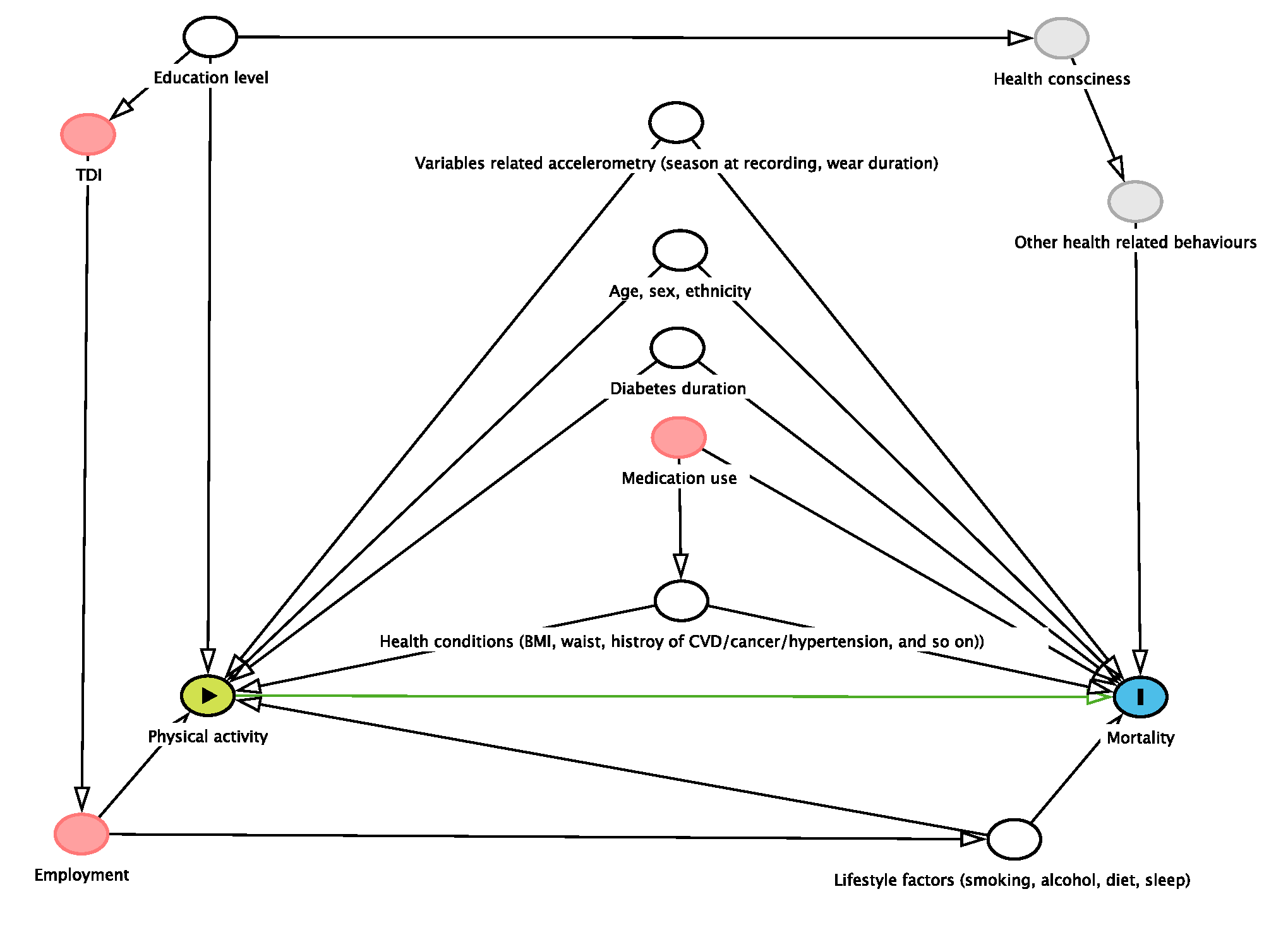


Green circle: exposure; blue circle: outcome; white circles: adjusted variables; pink circles: variables that do not need to be adjusted; grey circles: unobserved variables.

TDI, Townsend deprivation index; BMI, body mass index; CVD, cardiovascular disease.
