# Supplementary material for: Accelerometer-derived physical activity and mortality in individuals with type 2 diabetes
Source: Nat Commun. 2024 Jun 17;15:5164. doi: 10.1038/s41467-024-49542-0 (PMC11183112; doi:10.1038/s41467-024-49542-0)
Supplement: Supplementary file 3 — Reporting Summary [file 41467_2024_49542_MOESM3_ESM.pdf]

nature portfolio

Corresponding author(s): Dr. Chenjie Xu

Last updated by author(s): Apr 23, 2024

Reporting Summary

Nature Portfolio wishes to improve the reproducibility of the work that we publish. This form provides structure for consistency and transparency in reporting. For further information on Nature Portfolio policies, see our Editorial Policies and the Editorial Policy Checklist.

Statistics

For all statistical analyses, confirm that the following items are present in the figure legend, table legend, main text, or Methods section.

n/a	Confirmed	
☐	☒	The exact sample size (*n*) for each experimental group/condition, given as a discrete number and unit of measurement
☐	☒	A statement on whether measurements were taken from distinct samples or whether the same sample was measured repeatedly
☐	☒	The statistical test(s) used AND whether they are one- or two-sided<br>*Only common tests should be described solely by name; describe more complex techniques in the Methods section.*
☐	☒	A description of all covariates tested
☐	☒	A description of any assumptions or corrections, such as tests of normality and adjustment for multiple comparisons
☐	☒	A full description of the statistical parameters including central tendency (e.g. means) or other basic estimates (e.g. regression coefficient) AND variation (e.g. standard deviation) or associated estimates of uncertainty (e.g. confidence intervals)
☐	☒	For null hypothesis testing, the test statistic (e.g. *F*, *t*, *r*) with confidence intervals, effect sizes, degrees of freedom and *P* value noted<br>*Give P values as exact values whenever suitable.*
☒	☐	For Bayesian analysis, information on the choice of priors and Markov chain Monte Carlo settings
☒	☐	For hierarchical and complex designs, identification of the appropriate level for tests and full reporting of outcomes
☒	☐	Estimates of effect sizes (e.g. Cohen's *d*, Pearson's *r*), indicating how they were calculated

Our web collection on statistics for biologists contains articles on many of the points above.

Software and code

Policy information about availability of computer code

Data collection	All data in this study were derived from the UK Biobank.
Data analysis	All the analyses were conducted using STATA 16 statistical software (Stata Corp LLP, college station, TX) and R software (version 4.1.3). R packages: 'rms' (v6.7.1), 'mice' (v3.16.0), 'survival' (v3.5.7). The codes are available upon request from corresponding authors.

For manuscripts utilizing custom algorithms or software that are central to the research but not yet described in published literature, software must be made available to editors and reviewers. We strongly encourage code deposition in a community repository (e.g. GitHub). See the Nature Portfolio guidelines for submitting code & software for further information.

Data

Policy information about availability of data

All manuscripts must include a data availability statement. This statement should provide the following information, where applicable:

- Accession codes, unique identifiers, or web links for publicly available datasets
- A description of any restrictions on data availability
- For clinical datasets or third party data, please ensure that the statement adheres to our policy

The main data used in this study were accessed from the publicly available UK Biobank Resource (https://www.ukbiobank.ac.uk) under application number 79095, which cannot be shared with other investigators due to data privacy laws. The UK Biobank data can be accessed by researchers on the application. Source data are provided with this paper.

Research involving human participants, their data, or biological material

Policy information about studies with human participants or human data. See also policy information about sex, gender (identity/presentation), and sexual orientation and race, ethnicity and racism.

Reporting on sex and gender	The reported sex of participants was accessed through UK Biobank data-field 31, and included as a covariate in the statistical analyses. We conducted a subgroup analyses on sex and found that the association between LPA and all-cause mortality was stronger among female than male.<br>Gender was not considered in this study.
Reporting on race, ethnicity, or other socially relevant groupings	Participants' ethnicity was sourced from the UK Biobank data-field 21000 and incorporated as a covariate in the statistical evaluations. Participants self-identified their ethnic background during the baseline period (2006 - 2010), with further details available within the UK Biobank.
Population characteristics	19,624 diabetic participants with valid accelerometer data were left in the main analysis, with a mean age of 62.3 years (standard deviation, 7.8 years) and 44.4% were males. The baseline characteristics of 19,624 participants are shown in Table 1. Overall, participants who undertook more MVPA were generally younger with more females, lower BMI and waist circumference, higher diet scores, and shorter diabetes durations; were less likely to smoke or have a history of cancer, CVD, or hypertension.
Recruitment	The UK Biobank is a large population-based prospective cohort that recruited approximately 500,000 participants aged 40-73 years in the United Kingdom. Participants visited one of 22 assessment centers across England, Scotland, and Wales. 236,519 UK Biobank participants were invited to participate in an accelerometer study. Between 2013 and 2015, 240,000 invitations were sent to them for PA measurement by accelerometers, with a response rate of 44%. Devices were dispatched for 106,053 participants and, of these, data were received from 103,666.<br>Raw accelerometer data from 103,666 participants were further processed by the UK Biobank accelerometer expert working group. In addition, according to the exclusion criteria, 19,624 participants with valid data were finally included in the current study.
Ethics oversight	UK Biobank has approval from the North West Multi-centre Research Ethics Committee as a Research Tissue Bank (RTB) approval. This approval means that researchers do not require separate ethical clearance and can operate under the RTB approval. Informed consents were obtained from all participants. The ethical approval for the UK Biobank is extensively detailed and described online: https://www.ukbiobank.ac.uk/learn-more-about-uk-biobank/about-us/ethics. This current study was undertaken under project 79095.

Note that full information on the approval of the study protocol must also be provided in the manuscript.

Field-specific reporting

Please select the one below that is the best fit for your research. If you are not sure, read the appropriate sections before making your selection.

☒ Life sciences ☐ Behavioural & social sciences ☐ Ecological, evolutionary & environmental sciences

For a reference copy of the document with all sections, see nature.com/documents/nr-reporting-summary-flat.pdf

Life sciences study design

All studies must disclose on these points even when the disclosure is negative.

Sample size	No statistical methods were used to predetermine sample size. Firstly, we included 22,309 participants with T2D at baseline and accelerometry data. Exclusions were implemented for individuals who had insufficient wear time (n = 1,538), had dayight saving time shifts during wear period (n = 909), and had missing information on covariates (n = 238). Finally, 19,624 participants with T2D included in the primary analysis. These sample size are sufficient for the analyses according to the previous published studies using data from UK Biobank.
Data exclusions	The data exclusion criteria were documented in Methods and Supplementary Fig. 1. The exclusion criteria are as follows: 1) those who did not had T2D at baseline; 2) those who did not had device-measured PA data; 3) those who had insufficient wear time; 4) those who had daylight saving time shifts during wear period; 5) those who had missing information on covariates.
Replication	This is a population-based cohort study, and we have not yet replicated the findings in other samples. However, we used different statistical methods to verify our findings: 1) carefully controlling for a wide range of potential covariates; 2) excluding participants with cancer or CVD at baseline when assessing the association between PA and cause-specific mortality; 3) excluding participants with poor self-rated health status; 4) running landmark analyses; 5) performing stratifying analyses by the number of diabetes severity factors; 6) mutually adjusting for different intensities of PA; 7) repeating the main analysis after imputing the missing values of covariates using chained equation multiple imputations; 8) running competing risk analyses and so on (detailed in Methods and Results in the paper). Our main findings were rather robust and consistent across these analyses.
Randomization	This is an observational cohort study, and randomization is not applicable in this study. However, we carefully controlled for a wide range of potential covariates in the Cox regression models to mimic randomization.
Blinding	Blinding was not applicable to this study as this study is observational.

Reporting for specific materials, systems and methods

We require information from authors about some types of materials, experimental systems and methods used in many studies. Here, indicate whether each material, system or method listed is relevant to your study. If you are not sure if a list item applies to your research, read the appropriate section before selecting a response.

Materials & experimental systems

n/a	Involved in the study
☒	☐ Antibodies
☒	☐ Eukaryotic cell lines
☒	☐ Palaeontology and archaeology
☒	☐ Animals and other organisms
☒	☐ Clinical data
☒	☐ Dual use research of concern
☒	☐ Plants

Methods

n/a	Involved in the study
☒	☐ ChIP-seq
☒	☐ Flow cytometry
☒	☐ MRI-based neuroimaging

Plants

Seed stocks	NA
Novel plant genotypes	NA
Authentication	NA
